# NREM2 and Sleep Spindles Are Instrumental to the Consolidation of Motor Sequence Memories

**DOI:** 10.1371/journal.pbio.1002429

**Published:** 2016-03-31

**Authors:** Samuel Laventure, Stuart Fogel, Ovidiu Lungu, Geneviève Albouy, Pénélope Sévigny-Dupont, Catherine Vien, Chadi Sayour, Julie Carrier, Habib Benali, Julien Doyon

**Affiliations:** 1 Department of Psychology, University of Montreal, Montreal, Quebec, Canada; 2 Functional Neuroimaging Unit, C.R.I.U.G.M., Montreal, Quebec, Canada; 3 Department of Psychology, Western University, The Brain & Mind Institute, London, Ontario, Canada; 4 KU Leuven, Leuven, Belgium; 5 Center for Advanced Research in Sleep Medicine, Montreal, Quebec, Canada; 6 Sorbonne Universités, UPMC Univ Paris 06, CNRS, INSERM, Laboratoire d’Imagerie Biomédicale (LIB), Paris, France; Radboud Universiteit Nijmegen, NETHERLANDS

## Abstract

Although numerous studies have convincingly demonstrated that sleep plays a critical role in motor sequence learning (MSL) consolidation, the specific contribution of the different sleep stages in this type of memory consolidation is still contentious. To probe the role of stage 2 non-REM sleep (NREM2) in this process, we used a conditioning protocol in three different groups of participants who either received an odor during initial training on a motor sequence learning task and were re-exposed to this odor during different sleep stages of the post-training night (i.e., NREM2 sleep [Cond-NREM2], REM sleep [Cond-REM], or were not conditioned during learning but exposed to the odor during NREM2 [NoCond]). Results show that the Cond-NREM2 group had significantly higher gains in performance at retest than both the Cond-REM and NoCond groups. Also, only the Cond-NREM2 group yielded significant changes in sleep spindle characteristics during cueing. Finally, we found that a change in frequency of sleep spindles during cued-memory reactivation mediated the relationship between the experimental groups and gains in performance the next day. These findings strongly suggest that cued-memory reactivation during NREM2 sleep triggers an increase in sleep spindle activity that is then related to the consolidation of motor sequence memories.

## Abstract 2

Targeted reactivation of a motor sequence memory through re-exposure with a conditioned olfactory stimulus during the night reveals the specific role of stage 2 non-REM sleep and spindles in the consolidation process.

## Author Summary

There is ample evidence that sleep contributes to the consolidation (from a labile to a robust state) of a memory trace formed through learning of a new motor sequence, such as piano-playing. Sleep, however, is a complex neurophysiological state comprising several phases, and the role that each phase plays in this memory process remains controversial. To address this issue, we used a conditioning paradigm with human volunteers in which an odor was first associated (or not) with the practice of a motor sequence learning task. The odor was then presented again in one of two different sleep phases—rapid-eye movement (REM) or stage 2 non-REM (NREM2)—during the second half of the night, as a means of facilitating reactivation of the memory trace. As predicted, we found that participants cued with the odor during NREM2 showed greater gains in performance (reflecting better consolidation) the following morning compared with those who were cued during REM sleep or who had not previously been exposed to the odor during training. We also observed that reactivation during NREM2 sleep prompted significant changes in a type of brain activity known as sleep spindles. We conclude that NREM2 sleep—and spindles in particular—contribute critically to the consolidation of motor sequence memories.

## Introduction

From lacing up a shoe to typing at a computer, motor skills are learned and become automatic through the repetitive practice of a precise series of movements. This type of procedural memory, known as motor sequence learning (MSL), depends initially on repeated practice but is also enhanced offline after initial training. During this latent post-learning phase, the memory trace of a given motor experience is thought to be transformed into an enduring state, a process called memory consolidation [1–4]. A plethora of studies has shown that sleep is critical for consolidating the memory trace of newly acquired motor sequences, especially when the sequence of movements is known explicitly prior to the training phase [2,5–8].

While some authors investigating the existence of a relationship between sleep stages and the consolidation of motor memories using implicit [9,10] or explicit [11] sequential tasks proposed that consolidation was linked to rapid eye movement (REM) sleep, there is now increasing evidence that the consolidation of sequential motor memories is associated with changes in non-REM (NREM) sleep [7,12–15] and to alterations in characteristics of sleep spindles, in particular [16–20]. Sleep spindles are short (<2 s), synchronous bursts of activity between 11 and 17 Hz. They are found throughout NREM sleep but are particularly numerous in NREM stage 2 (NREM2) sleep. Importantly, animal studies have shown that neurons activated during a motor task are reactivated during subsequent NREM sleep [21] and that these task “replays” during sleep coincide with slow-wave and spindle events [22]. Although a link between sleep spindles and consolidation of sequential motor learning has also been observed in many experiments in humans (for review, see [23]), most findings reported so far have been correlational in nature, hence precluding the determination of whether their role in consolidation is causal or not.

To address this limitation, researchers have recently investigated the effects of manipulating sleep-specific brain oscillations (e.g., slow oscillations [24]) or neurotransmitter systems (e.g., noradrenaline [15]) in order to probe the tenets of reactivation and consolidation mechanisms of memory, thereby getting closer to testing for causality [23,25,26]. Others have employed conditioning experimental designs, also called targeted memory reactivation (TMR) paradigms, which are thought to target the processing of specific memory representations during sleep through reactivation with an external olfactory or auditory stimulus that was previously associated during training [27]. The use of this innovative technique has demonstrated that it is possible to enhance performance on declarative [28–31], complex/cognitive procedural [32], and, more recently, MSL tasks [33–35] by cuing subjects during their sleep with olfactory [29,30,36] and/or auditory stimuli ([25,28–33]; see reviews [25–27]).

More specifically in the procedural memory domain, the TMR approach has been employed in four studies to investigate the contribution of specific sleep stages in the consolidation of MSL. First, in a seminal study, Rasch and colleagues [29] used a rose-like odor as a context cue while participants performed two successive tasks (visuospatial and MSL) and re-exposed subjects to the olfactory cue either during SWS or REM sleep. They hypothesized that re-exposure to the olfactory stimulus in REM sleep would improve consolidation of this type of learning. Contrary to their prediction, however, subjects did not show any difference in motor memory consolidation after re-exposure to either sleep stage. Although conjectural, one reason for the lack of a significant effect may be that subjects were cued during SWS and REM, but not during NREM2 sleep. As sleep spindles are thought to be implicated in the consolidation of MSL, it is possible that an improvement in post-sleep performance on the motor task might be observed if the conditioned stimulus is presented instead during NREM2, a sleep stage during which spindles occur most often [37–39]. Since the same odor was used as stimulus for both types of task, it is also possible that the context-association of one task interfered with the other, hence reflecting the presence of improvements on the declarative but not the MSL task.

Other investigators have used tones and melodies associated to the learned motor task as cues for memory reactivation during the post-learning night, and have demonstrated overnight improvement in performance on an MSL task the morning after [33–35]. In the latter three studies, procedural learning as well as enhanced motor sequence consolidation were assessed using versions of the serial reaction-time task (SRTT), and auditory cuing condition was administered either during slow-wave sleep (SWS) [34,35] or during the night without distinction to the sleep stage [33]. Overall, their results revealed that re-exposure to auditory cues associated with learning during sleep, and more precisely SWS, improved motor memory consolidation. This boosting in performance was also specific to cued-memory reactivation performed during sleep [33,35] and, remarkably, to the finger transitions that were cued [33], as no performance enhancement was observed when cued-memory reactivation occurred during a wake period [33,34], when part of the sequence was not cued [33], or when subjects were tested in a no-cuing condition [33,35]. Importantly, correlations were also found between sleep spindles ([31–32], but see [33] for a different pattern of findings), and gains in performance on the sequence task, suggesting again that sleep spindles play a role in the reactivation of the motor memory trace. Yet despite the fact that these TMR studies provide correlational evidence of a link between sleep spindles and motor sequence consolidation, none have reported changes in spindle activity, which were then associated with better motor memory consolidation. Furthermore, in both studies that found correlations between sleep spindles and performance, cuing of the conditioned stimuli was carried out during SWS, and not NREM2 sleep. If sleep spindles are crucial in the consolidation process, one would expect that cuing during NREM2 sleep would be most effective in modulating spindles, irrespective of the type of conditioned stimulus. Finally, only one of the previous studies using a TMR approach compared cuing conditions in two different sleep stages, hence limiting somewhat one’s interpretations regarding the specificity of the sleep stage during which reactivation of the memory trace optimizes motor sequence consolidation.

**Fig 1:**
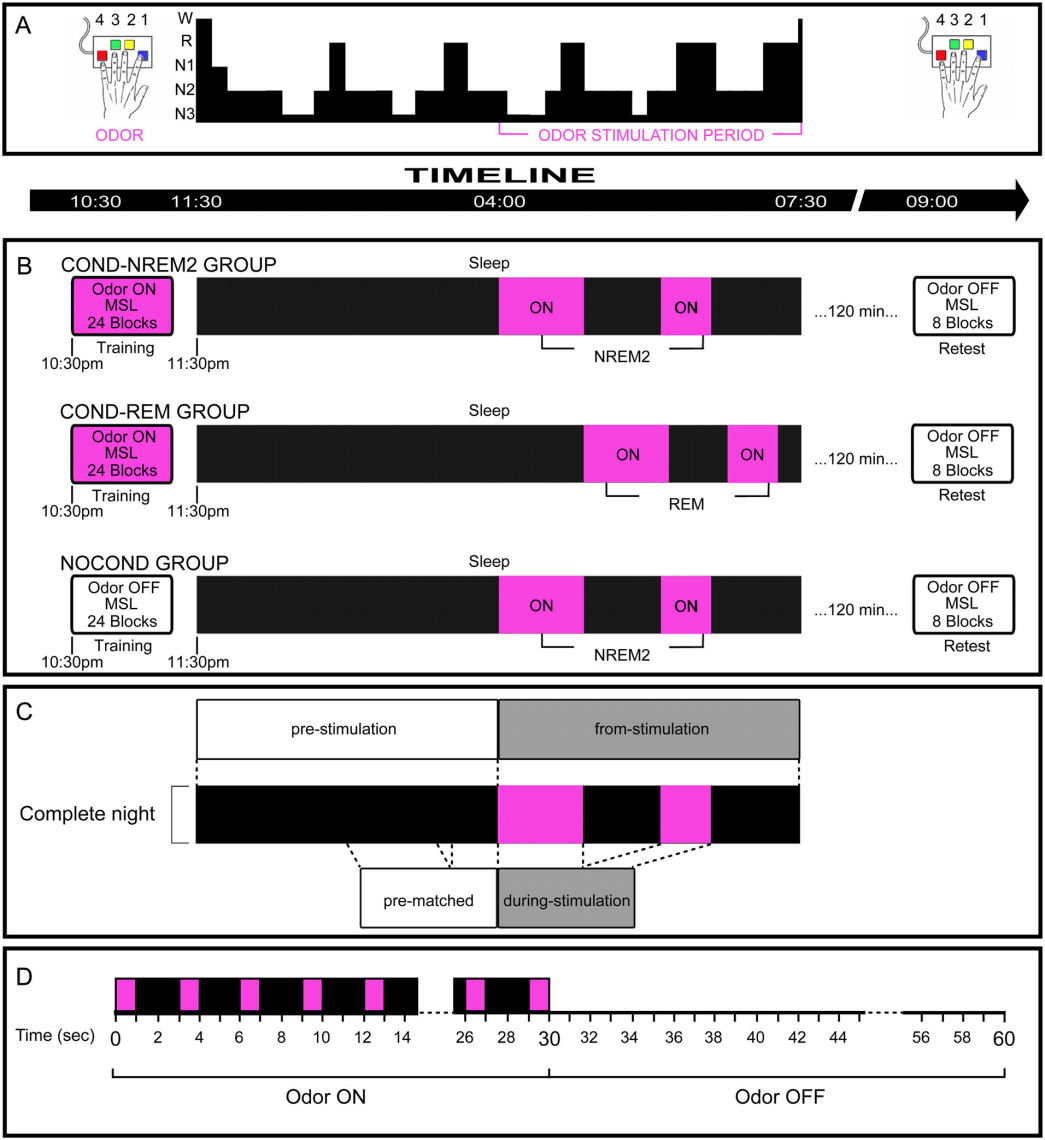
Experimental design. (A) **Overview of the experimental design**. The odor was first presented while participants performed the MSL task. They were then re-exposed during sleep to the associated olfactory cue. This type of manipulation, called targeted memory reactivation (TMR), is thought to reactivate part of the memory trace previously associated to the cue. The effect of the manipulation was assessed by comparing performance between the evening training and morning retest sessions. (B) **Experimental groups, exposure, and cueing protocol**. Subjects were randomly assigned to one of three groups. Both Cond-NREM2 and Cond-REM groups were exposed to the odor during the evening training session and re-exposed to the same stimulus during their respective sleep stage. By contrast, the NoCond group wasn’t exposed to the odor while training, but received olfactory stimulation during NREM2 sleep. All groups were exposed to the odor during the second half of the night and were retested the next morning. (C) **Description of the segmentation of sleep periods**. The Pre-stimulation and from-stimulation periods were defined for each participant using the onset of exposure to the odor during sleep. The during-stimulation period represented the period during which the odor was presented to the participants while in their target sleep stage. The pre-matched period consisted of a period of sleep that corresponded to the exact same length as that of the during-stimulation period, and that occurred just before the onset of the olfactory cuing. (D) **Olfactory delivery method.** Odor delivery followed an ON/OFF block design. During ON blocks, the odor was sent during 1 s (in pink) every 3 s, while OFF blocks consisted of periods without odor delivery. For the MSL training session, the ON blocks consisted of the period during which subjects were practicing the sequence, while the OFF blocks corresponded to the periods of 30 s of rest in-between. During the targeted stage of sleep, the odor was delivered on a 30 s ON/30 s OFF block design for a maximum of 60 min.

In the present study, we thus investigated the contributing role of NREM2 sleep—in particular, via the action of sleep spindles—on MSL using an olfactory TMR paradigm design similar to the one used by Rasch et al. [29]. Following an MSL training session in which we exposed participants to a rose-like odor, subjects were then re-exposed to the same olfactory stimulus during NREM2 or REM sleep occurring during the second half of the post-learning night (see Fig 1A). This approach did not only enable us to compare the effect of these two olfactory stimulation conditions during similar portions of the night, but also to compare sleep spindle activity before and after stimulation in order to test directly whether cuing resulted in an increase in sleep spindles and their characteristics (e.g., amplitude, duration, frequency, and density) associated with performance gains. We also tested a third group that was not conditioned to any odor during training, but was exposed for the first time to the odor during NREM2. The latter group allowed us to ensure that our results would not be biased due to changes in NREM2 sleep and spindle characteristics induced by mere exposure to the olfactory stimulation during sleep.

Each group was retested in the morning to measure the subjects’ level of consolidation as reflected through gains in performance. We conjectured that each group would consolidate the MSL task during sleep as shown by gains in performance during the retest session. In addition, we hypothesized that cuing in the post-training night to a conditioned olfactory stimulus during NREM2 sleep, as compared to re-exposure during REM or exposure during NREM2 with no prior conditioning, would produce greater gains in performance on the MSL task the next day, hence demonstrating that re-exposure to the conditioned stimulus associated with the motor memory trace during NREM2 enhanced the consolidation process. Finally, consistent with the literature showing that sleep spindles are involved in motor memory consolidation, we proposed that re-exposure to the conditioned olfactory stimulus during NREM2 sleep would significantly increase spindle activity for the group conditioned and re-exposed during NREM2 sleep only.

## Results

### MSL Consolidation as a Function of Odor Manipulation

**Fig 2:**
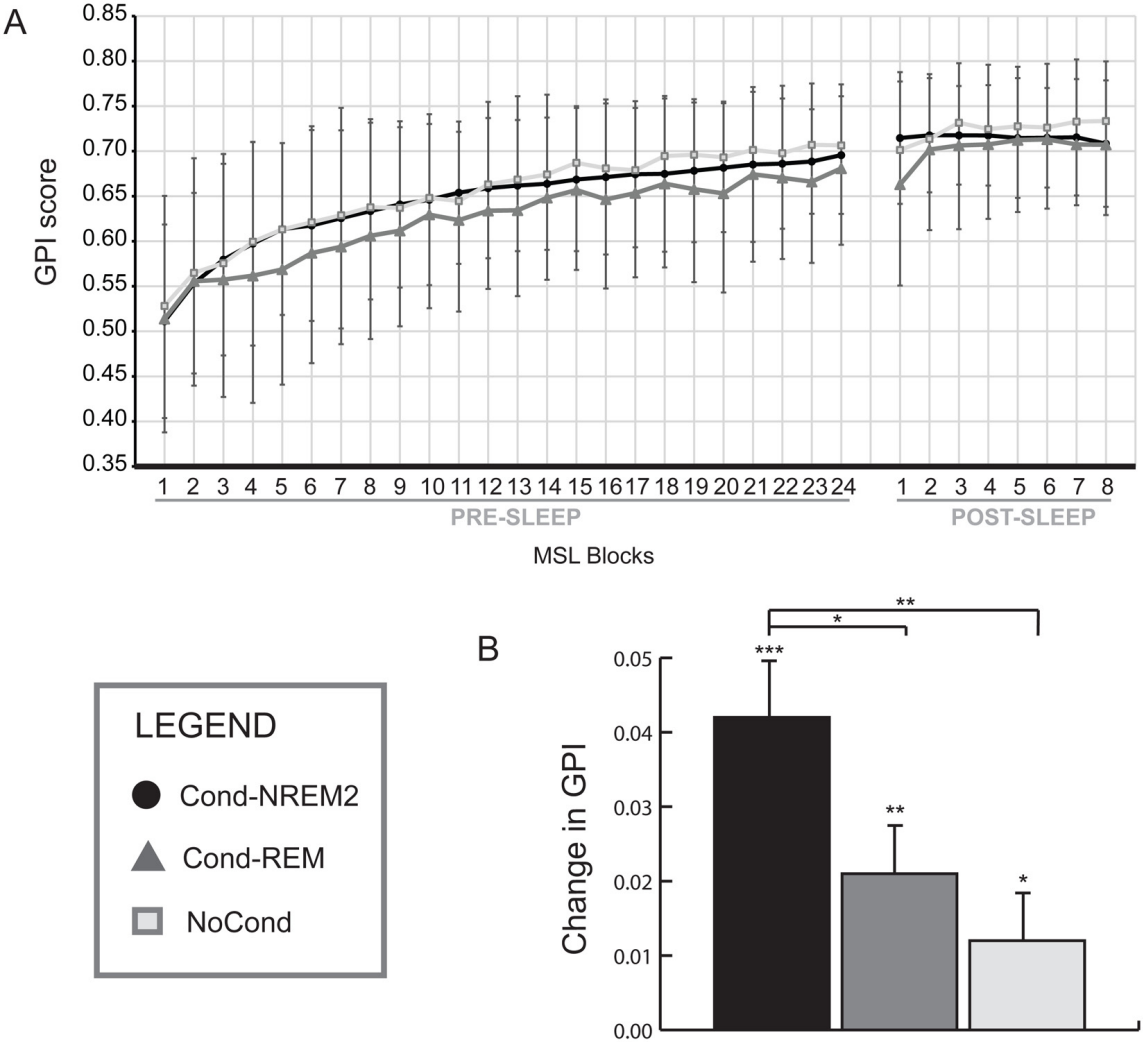
Behavioral results. (A) **MSL learning curves**. Learning curves of all three groups during the evening and morning MSL sessions. Scores were calculated with the global performance index (GPI). Blocks used for the calculation of the change in GPI (i.e., offline gains) are indicated on the *x*-axis in bold format. (B) **Offline gains in performance on the MSL task**. This graph illustrates the offline gains per group on the MSL task performance as measured by the mean GPI between the four first blocks of post-sleep retest and the four last blocks of pre-sleep training sessions. All groups showed increases in performance after a night of sleep. The Cond-NREM2 group had significantly higher gains than both the Cond-REM and NoCond groups. No difference was found between the Cond-REM and NoCond groups. Data deposited in the Dryad repository: http://dx.doi.org/10.5061/dryad.b4t60 [40]. * *p* < 0.05; ** *p* < 0.01; *** *p* < 0.001

Offline gains in performance in the MSL task were assessed in 76 participants using a global performance index (GPI) corresponding to a measure of performance that takes into account possible speed and accuracy trade-offs (Fig 2A; see S2 Fig for detailed information on separate measures of speed and accuracy).

A mixed repeated measures ANOVA of the GPI performed on the entire session of training (24 blocks—repeated measures) showed a significant effect of block (F_23, 1679_ = 77.771, *p* < .00001), with no significant block x group interaction (F_46, 1679_ = .422, *p* = .99) nor any main effect of group (F_2, 73_ = .785, *p* = .46). These results suggest that, while all participants showed improvement in performance across the training session, all groups had similar rates of learning and overall level of motor performance. Furthermore, a similar analysis performed on the last four blocks of the evening training session only revealed no significant main effect of block (F_3, 219_ = .423, *p* = .42), block x group interaction (F_6, 219_ = .623, *p* = .71), or between subjects effect of group (F_2, 73_ = 1.338, *p* = .27), showing that, at the end of the training session, all participants had reached an asymptotic performance and that the performance level in the MSL task was similar among the three groups.

Differences in level of consolidation were measured by comparing the mean GPI score of the first four blocks of retest to the last four blocks of training using a repeated measures ANOVA while controlling for individual cuing duration and olfactory threshold. The results yielded a main effect of session (F_1, 71_ = 49.901, *p* < .00001) and a session x group interaction (F_2, 71_ = 5.794, *p* = .005), hence demonstrating that while all participants revealed gains in performance between the two sessions, there was a difference in the level of motor skill consolidation between groups. As predicted, post-hoc univariate tests demonstrated that each group significantly increased their performance across sessions (Cond-NREM2: mean = .042 ±.012, *p* < .00001; Cond-REM: mean = .021 ± .013, *p* = .001; NoCond: mean = .012 ± .012, *p* = .04; see Table 1). Most importantly, however, planned contrasts analyses revealed that the Cond-NREM2 group exhibited significantly higher gains in performance than both the Cond-REM (*p* = .03) and NoCond (*p* = .001) groups, and that Cond-REM and NoCond groups did not differ significantly (*p* = .27), demonstrating that cued-memory reactivation during NREM2 enhanced performance over-and-above the gains normally afforded by sleep.

**Table 1:** Offline GPI gains on the MSL task.

	*n*	GPI	95% CI	*p*
**All subjects (*n* = 76)**				
**Cond-NREM2**	25	.042	.012	< .00001
**Cond-REM**	23	.021	.013	0.001
**NoCond**	28	.012	.012	0.04
**PSG subset (*n* = 64)**				
**Cond-NREM2**	21	.041	.033	< .0001
**Cond-REM**	21	.019	.014	0.008
**NoCond**	22	.010	.014	0.17

Results from the ANOVA for repeated measures assessing the level of offline gains in performance (consolidation) between the evening and morning MSL sessions as measured with the GPI for each of the experimental groups. Analyses from all subjects and the polysomnographic (PSG) subset are shown. These results demonstrate that all groups in the main set of participants (all subjects) showed a significant increase in performance after a night of sleep.

In order to relate more directly the subjects’ level of motor sequence consolidation with their own polysomnographic (PSG) data, we then carried out additional behavioural analyses after discarding 12 participants in whom electroencephalography (EEG) recordings were of poor quality because of technical difficulties. The analyses based upon this smaller subset of subjects (i.e., *n* = 64) yielded a similar (albeit not identical) pattern of results. While the overall distribution of group means was similar, the overnight offline gains in performance differed slightly as changes in performance in the Cond-NREM2 (mean gain = +.041 ± .033, *p* < .0001) and Cond-REM (mean gain = +.019 ± .014, *p* = .008) groups remained significant, but those related to the NoCond group did not reach significance (mean gain = +.010 ± .014, *p* = .17). Because the NoCond group (from the “all subjects” set) demonstrated the smallest overnight MSL consolidation effect (*p* = 0.01), it is not surprising that a subset of this group (i.e., the PSG subset) did not reach significance using the same measure. More importantly, however, when looking at both sets of subjects, the significant differences in offline gains between groups did not change, hence demonstrating that the cuing procedure was effective. Finally, it is important to note that the pattern of results described above could not be due to poor control of olfactory cuing because stimulation in targeted sleep stages was successful (see S1 Fig). Furthermore, the present findings cannot be due to the rejection of subjects with outlying performance (see Method below), as additional behavioral analyses including those individuals (*n* = 4) revealed similar results (see S1 Text).

### Olfactory Threshold and Stimulation during Sleep Analyses

A one-way ANOVA showed that there were no differences between groups in olfactory detection threshold (F_2, 73_ = 2.324, *p* = .10) as measured with the Sniffin’sticks test. The same analysis performed on the participants included in the PSG analyses also produced similar results.

Another series of analyses were conducted to ensure that the length of exposure to the rose-like odor during sleep did not differ between groups, and that targeted stages were successfully stimulated. First, a one-way ANOVA revealed that there was no difference in the total length of exposure to the odor cue during sleep between the three groups (F_2, 73_ = 2.671, *p* = .08; Cond-NREM2: 53.4 min ± 3.3, Cond-REM: 48.4 min ± 3.4, NoCond: 49.2 min ± 3.1). Then, another one-way ANOVA testing for differences during NREM2 was conducted (F_2, 73_ = 73.127, *p* < .0001), and post-hoc univariate tests demonstrated that there was no difference in the duration of exposure to the odor during NREM2; that is between the Cond-NREM2 (39 min ± 4.4 min) and NoCond groups (32 min ± 4.2 min) (see S1 Fig and S1 Table for detailed analyses).

### Sleep Architecture

Sleep architecture and spindles were analyzed using recordings throughout the night, which were separated into several distinct periods (see Methods for details): (a) sleep occurring before onset of the olfactory stimulation (pre-stimulation), (b) sleep following the start of olfactory stimulation (from-stimulation), (c) sleep during stimulation in the targeted sleep stage (during stimulation), and (d) sleep of the same length as during-stimulation, but selected in the pre-stimulation period (pre-matched) (see Fig 1C).

One-way ANOVAs performed separately on pre- and from-stimulation periods did not reveal any significant difference between the three groups in any of the sleep stages with regards to the total sleep time (TST), total recording time (TRT), sleep efficiency (SE), or wake duration, demonstrating that our manipulation did not generate differential changes in sleep architecture between our groups (see S2 Table for more information).

### Sleep Spindle Analyses

Spindles were extracted from Fz, Cz, and Pz derivations with an automatic algorithm [41]. Sleep spindle analyses focused on peak amplitude, duration, peak frequency, and density. Spindle analyses were only conducted in the Cond-NREM2 and NoCond groups, as no spindle were generated during the Cond-REM targeted stage. All analyses were carried on NREM2 sleep spindles only.

A categorization algorithm was used to identify spindles originating from a single source (e.g., a unique channel/electrode—see Method section). Overall, the algorithm identified 29.8% of spindles at Fz, 36.2% at Cz, and 20.1% at Pz, as overlapping spindles. Filtering of these redundant spindle events had the effect of lowering the median spindle frequency value of the overall spindle distribution from 11.49 Hz to 11.37 Hz at Fz (t_63_ = -5.439, *p* < .001) and increasing the median frequency from 13.40 Hz to 13.60 Hz at Pz (t_63_ = 3.131, *p* = .003). Thus, this categorization approach helped to reduce the overlap between frontal and parietal distribution of spindle frequencies, hence allowing for a better classification between these two midline sources. All analyses of sleep spindle characteristics reported below were thus carried out using these filtered events. Yet note that a similar pattern of results was observed when analyses included the entire set of spindles before applying the categorization algorithm (see S1 Text).

#### Fz and Cz derivations

There were no significant differences between the two groups in terms of peak amplitude, duration, peak frequency, and density of sleep spindles in the pre-matched and during-stimulation periods or when looking at the difference between during-stimulation and pre-matched periods in both frontal (Fz) and central regions (Cz).

#### Pz derivation

A one-way ANOVA did not reveal any statistically significant differences between the two experimental groups with respect to spindles amplitude, duration, frequency, and density in the pre-matched period, suggesting that, before stimulation, spindles characteristics were similar between groups. No difference was found in the during-stimulation period, either (see S3 Table).

**Fig 3:**
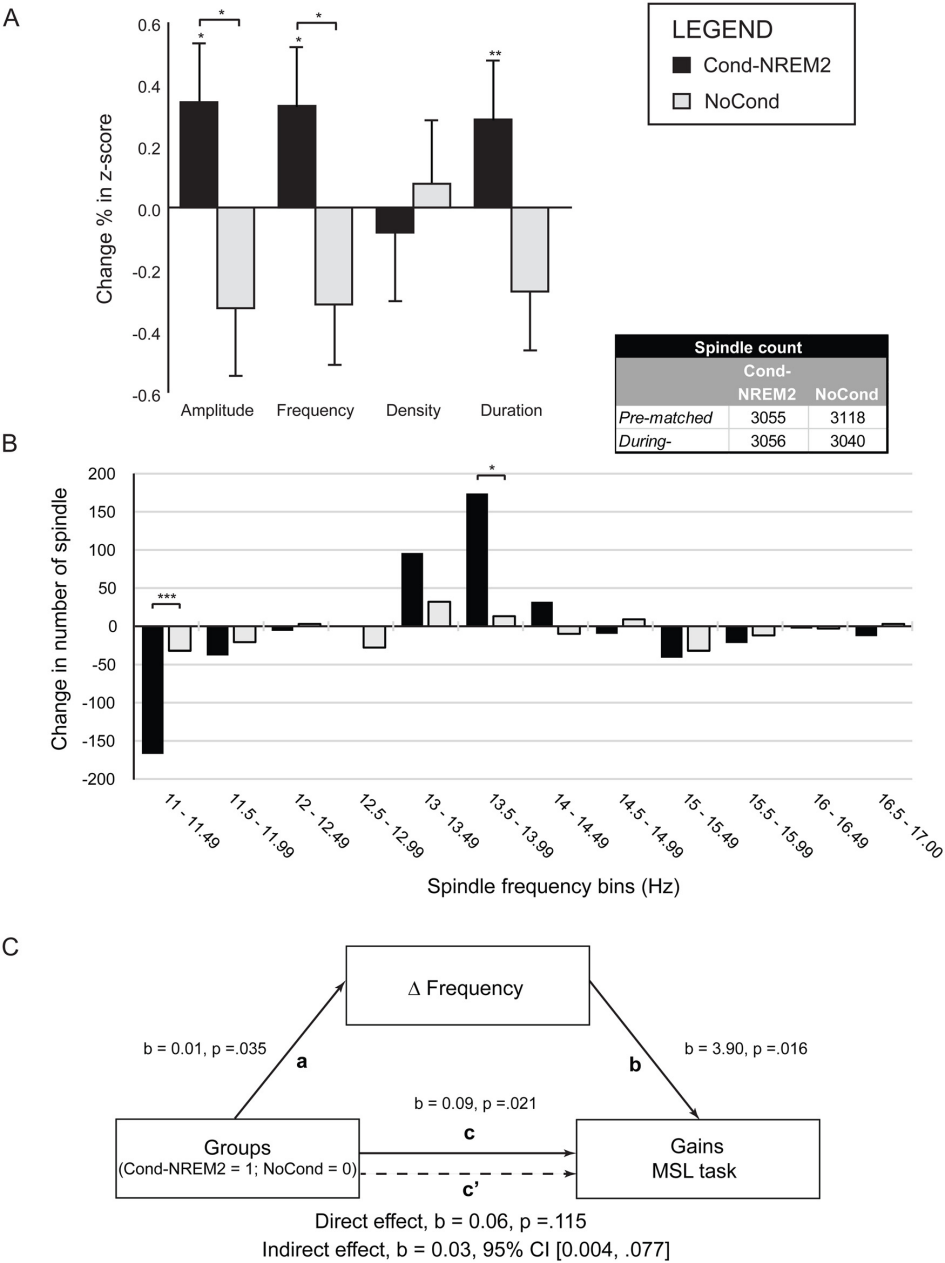
Sleep spindle results. (A) **Changes in parietal sleep spindle characteristics**. Standardized (Z-score) differences in sleep spindle characteristics between the pre-matched and during-stimulation periods at Pz. Only the Cond-NREM2 group showed significant increase in spindle amplitude, frequency, and duration. Amplitude and frequency were significantly different between the Cond-NREM2 and NoCond groups. (B) **Changes in the number of spindles at Pz in specific frequency ranges.** Differences at Pz in the number of spindles categorized by frequency range between the pre-matched and during-stimulation sleep periods. Significance was determined using a Chi^2^ analysis with Bonferroni correction on the number of bins. The total number of spindles detected for each group and sleep period is shown in the “Spindle count” table. The results revealed a significant decrease of spindle in the 11–11.49 Hz range, but an increase in the 13.5–13.99 Hz range. (C) **Changes in frequency at Pz mediates the relationship between the TMR protocol and MSL offline gains**. The significant relation between the experimental protocol and the gains in performance on the MSL task (relation c) disappeared when the change in frequency in sleep spindles over the parietal cortex between the pre-matched and *during-stimulation* periods were included in the mediation model (direct effect: relation c’). The indirect effect composed of (1) the experimental protocol and the change in spindle frequency (relation a) and (2) the change in spindle frequency and the MSL offline gains (relation b) was significant, as demonstrated by the bootstrap analysis (Cl .004, .077). This pattern of results strongly suggests that sleep spindles occurring in the parietal regions are crucial to motor memory consolidation through an increase of spindles of higher frequency. Data deposited in the Dryad repository: http://dx.doi.org/10.5061/dryad.b4t60 [40]. * *p* < 0.05; ** *p* < 0.01; *** *p* < 0.001

By contrast, when we compared changes in spindle characteristics between the pre-matched and during-stimulation sleep periods, the one-way ANOVA comparing percent change (Δ%) revealed a significant difference in peak amplitude (F_1, 41_ = 5.257, *p* = .03) and peak frequency (F_1, 41_ = 4.842, *p* = .03) between the Cond-NREM2 and NoCond groups (see Table 2 for details). A similar pattern of results was observed for spindles duration, although the effect did not reach significance (F_1, 41_ = 3.523, *p* = .07). Follow-up, one-sample *t* tests revealed that only the Cond-NREM2 group had a significant increase in Δ% in peak frequency (Cond-NREM2: t_20_ = 2.443, *p* = .02; NoCond: t_21_ = -.511, *p* = .62), Δ% in peak amplitude (Cond-NREM2: t_20_ = 2.394, *p* = .03; NoCond: t_21_ = -.481, p = .64), and Δ% in duration (Cond-NREM2: t_20_ = 3.013, *p* = .007; NoCond: t_21_ = .881, *p* = .39) (Fig 3A).

**Table 2:** Differences in spindle characteristics between pre-matched and during-stimulation sleep periods.

	Cond-NREM2 (*n* = 21)			NoCond (*n* = 22)			F_(1, 42)_	*p*
	Δ%	T_(20)_	*p*	Δ%	T_(21)_	*p*		
Amplitude	**11.6%**	**2.394**	**.03**	-1.5%	-0.481	.64	**5.257**	**.03**
Frequency	**0.7%**	**2.443**	**.02**	-0.1%	-0.511	.61	**4.842**	**.03**
Density	0.6%	0.246	.81	2.3%	1.033	.31	0.261	.61
Duration	**10.1%**	**3.013**	**.007**	2.2%	0.881	.39	3.523	.07

One-sample *t* tests were carried out on spindle characteristics Δ% in each group. The results revealed that the stimulation probed an increase in amplitude, duration, and frequency of spindles in the Cond-NREM2 group, but not in the NoCond group. One-way ANOVAs tested for differences in the same characteristics between groups. Compared to NoCond, the Cond-NREM2 group spindles increased significantly in amplitude and frequency. Statistical significance is highlighted in bold.

Additionally, bootstrap analyses (with 5,000 samples) performed on changes in sleep spindles characteristics between the pre-matched and during-stimulation sleep periods at Pz showed that amplitude (*p* = .0012), frequency (*p* = .0026), and duration (*p* = .0094) yielded significant changes, while density (*p* = .7764) did not (see S3 and S4 Figs and S1 Text). Bonferroni correction for 12 comparisons (3 electrodes x 4 characteristics; reference *p*-value = .0041) applied on these results confirm the effects reported above: spindle amplitude and frequency increased significantly, while duration and density did not, between these two sleep periods.

To determine the precise spindle frequencies that were enhanced through cuing when comparing the pre-matched and during-stimulation periods, further Chi^2^ analyses were carried out on the change in number of spindles in each 0.5 Hz frequency bin from 11 Hz to 17 Hz. Such analyses revealed that very narrow ranges in spindle frequencies were modulated by the olfactory manipulation during sleep. Indeed, the number of slow frequency spindles for the Cond-NREM2 group in the 11 Hz to 11.49 Hz bin decreased (Χ^2^ [1, N = 1,109] = 18.04, *p* = 0.0003, bonf.-corrected), while the number of higher-frequency spindles in the 13.5 Hz to 13.99 Hz bin increased significantly (Χ^2^ [1, N = 2,463] = 18.04, *p* = 0.03, bonf.-corrected) compared to the NoCond group. Yet the total number of spindles (11–17 Hz) was similar between groups and periods of sleep (Fig 3B).

To ensure that the odor sent for a few minutes during SWS did not significantly influence the performance of the cued group, we tested if the duration of exposure to the odor in SWS at Pz correlated with gains in performance. Neither the results of the Cond-NREM2 nor the NoCond groups showed any significant correlations between the duration of stimulation in SWS and gains in performance (Cond-NREM2: r = -.168, *p* = 0.47; NoCond: r = -.012, *p* = 0.96). Also, we found no correlation between spindle characteristics and gains in performance in the Cond-NREM2 group (amplitude: r = 0.06, *p* = 0.82; frequency: r = -0.09, *p* = 0.73; density: r = -0.07, *p* = 0.78; duration: r = -0.16, *p* = 0.53).

#### Mediation analyses

Using NREM2 Δ% peak frequency at Pz from pre-matched versus during-stimulation as a mediator (see Fig 3C), we found a significant indirect effect of the experimental groups (Cond-NREM2 [1]; NoCond [0]) on gains in performance through Δ% peak frequency (*b* = 0.027 Bca CI [.004, .077]) with a medium effect size (*k* ^2^ = .106, 95% BCa CI [.018, .256]). Although differences were found between the Cond-NREM2 and NoCond groups in spindle amplitude, mediation analyses did not reveal a significant effect.

## Discussion

The present study investigated the contributing role of NREM2 and sleep spindles in motor sequence memory consolidation. As expected, all experimental groups showed offline improvements in their performance on the motor sequence task after a night of sleep, highlighting again the influence of sleep on the consolidation of this type of motor learning. More importantly, however, the TMR paradigm allowed us to demonstrate that post-training cuing during NREM2 sleep with a conditioned olfactory stimulus significantly increased sequential motor performance, while cuing during REM sleep or exposing the participant to an unconditioned stimulus during NREM2 did not. Our results also show that cuing during NREM2 sleep produced an increase in a variety of spindle characteristics, and that such changes were found only over parietal areas. Finally, we demonstrate that the increase in sleep spindle frequency over parietal regions mediated the difference between the MSL performance of the conditioned versus unconditioned group. Altogether, the results of the present study reveal that NREM2 sleep is crucial for sequential memory consolidation, and that part of this effect may be explained by a specific modulation of sleep spindles.

As expected, sleep had a positive impact on MSL consolidation in all three groups. The fact that the non-conditioned control group improved at retest is consistent with a large number of studies that have reported that a night of sleep is sufficient to trigger a consolidation process, which in turn produces gains in performance ([6,13,19,23]; but see [42] for a discussion of possible confounding factors). Furthermore, our pattern of results demonstrates that stimulation with the conditioned stimulus during REM sleep does not elicit greater behavioural improvement over and above the effect of sleep. This replicates the findings from Rasch et al. [29], who did not report any significant changes in performance on a very similar TMR protocol using the motor sequence learning task after presentation of a conditioned olfactory stimulus during REM or SWS. Although findings from Rasch et al. [29] and ours do not appear to support prior studies, which suggested that the process mediating motor sequence consolidation is dependent upon REM sleep [11,43,44], we argue that it was likely because of cognitive demands associated to the task employed (i.e., probabilistic serial reaction time task, mirror tracing task) and that it underlines the possibility that REM sleep might be implicated in other aspects (i.e., more cognitively demanding processes) of motor memory consolidation. Yet our results and those from other recent investigations [7,15–20] indicate that the consolidation of a newly acquired explicit and cognitively simple motor sequential skill is particularly dependent on NREM sleep.

### NREM2 Sleep Promotes Motor Memory Consolidation

More importantly, and as expected, our results show that participants conditioned to an olfactory stimulus during training to a novel MSL task, and re-exposed to that same stimulus during NREM2 sleep, experienced a significant enhancement in performance the next day compared to the two other groups. The latter finding is in accord with previous studies, which found that performance at retest on an MSL task is correlated to the amount of NREM [10] and to NREM2 sleep more specifically [13,20]. It is also consistent with evidence that pre-sleep training on a motor task increases the time spent in NREM2 sleep [45,46], but that deprivation of post-training SWS or REM sleep does not produce any decrease in motor task performance at retest [15,47,48], hence supporting further the conclusion that NREM2 sleep plays an important role in the motor sequence memory consolidation process (see [23] for a review).

It is important to note that previous TMR studies that investigated the implication of sleep in MSL learning found greater gains in performance when subjects were cued during SWS [34,35]. The current study extends these findings and provides, to our knowledge, the first evidence that possible reactivation of the motor memory trace during NREM2 sleep does facilitate offline consolidation processes capable of producing increases in sequential motor performance when tested again the next day. Our results are also in line with a recent integrative model proposed by Genzel and colleagues [14], which states that such memory trace reactivations occur particularly (but not exclusively) during the so-called light NREM sleep. Although still conjectural, this model put forward the notion that such an active memory consolidation process, possibly due to exchange of information between the striatum and motor related-cortical regions [49,50], would take place during this light NREM phase because it offers the most optimal conditions for global brain interactions. Thus consistent with Genzel’s model, our behavioral results strongly suggest that mnemonic processes occurring during NREM2 sleep are involved in sequential motor memory consolidation.

### Changes in Spindle Characteristics Associated with Motor Sequence Consolidation

Over and above the changes in performance as a result of cueing during NREM2, the present olfactory TMR protocol induced changes in sleep spindle characteristics (i.e., amplitude, duration, and frequency) only for the group conditioned and re-exposed during NREM2 sleep. Importantly, we demonstrate that these changes were not caused by differences in sleep architecture or spindles present prior to the stimulation, but were prompted specifically through re-exposure to the conditioned odor during NREM2 sleep. Previous TMR studies investigating the role of SWS in motor memory consolidation have reported correlations between gains in performance and the number or density of sleep spindles, but no change in spindle characteristics per se [34,35]. The reasons for such differences with our own findings may be two-fold. First, it is possible that because sleep spindles are more numerous in the stage we targeted (NREM2) than in SWS, predominance of spindles between these two stages could account for the increases in several characteristics shown in our own study. Second and most importantly, however, the protocol employed here offered the unique opportunity to compare the effect of reactivation during cuing compared to a baseline period recorded during the same night prior to any re-exposure. Thus, this enabled us to compare non-cued segments that were uncontaminated by previous cuing trials to cued segments of NREM sleep, allowing us, for the first time, to detect changes in sleep spindle characteristics in a TMR study on MSL consolidation.

Interestingly, the present study also revealed that re-exposure to the conditioned stimulus produced significantly greater increases in sleep spindle amplitude and frequency, and that this change was present at Pz for the group cued during NREM2, compared to the non-conditioned group. These results are in line with reports, as those from Schabus and colleagues [51–53], suggesting that changes in spindle characteristics may be more important than an increase in spindle density for consolidating memories. They are also in accord with some known properties related to the underlying physiology of sleep spindle oscillations. Indeed, a recent study demonstrated that sleep spindles of higher frequency had higher amplitude and, interestingly, greater rate of cortical propagation [54]. Since sleep and spindle burst activity are thought to provide favourable conditions for synaptic plasticity ([21,55–57], see [58] for a review), it is thus possible that, without the need for a larger number of spindle events, the increases in frequency and amplitude found in the present study allowed for a more efficient potentiation of long-term synaptic changes and increased synchrony in the thalamocortical loop, hence facilitating consolidation of the memory trace.

Further analyses on spindle frequencies at Pz showed that, in the conditioned NREM2 group only, the number of spindles increased in a distinct range of frequency (13.5–13.99 Hz), while there was a decrease in a lower frequency range (11–11.49 Hz). This pattern of results is even more revealing that the NoCond group did not show any change in either of the frequency bins, but revealed instead an overall normal pattern of decrease in mean frequency (S3 Table) that is usually observed during an undisturbed night of sleep in normal subjects [59]. In sum, since parietal spindles in the 13.5 Hz to 13.99 Hz range are generally considered fast spindles, our results in the Cond-NREM2 group are thus consistent with previous studies that have reported increases in fast spindle density [18] and higher sigma band (13 Hz) activity [19] over parietal regions following MSL training.

The reason why changes in spindle characteristics were found in the parietal regions only is speculative at this point. Yet it is now known that the early stage of MSL is characterized by the acquisition of two different sequence representations (motor, spatial), which are associated with plastic changes in different motor-related networks and that are depending differently on sleep for consolidation [60,61]. Learning the motor representation of the sequence relies, in part, on activation of the striatum, motor cortex, and cerebellum (see reviews [4,12,62,63]). By contrast, acquisition of the spatial representation depends mainly upon the hippocampus as well as the prefrontal and parietal regions [60,63,64]. Results from studies in our laboratory have demonstrated that the consolidation of the latter type of memory trace is related to spindle activity during NREM sleep [65]. Furthermore, TMR studies investigating the effect of sleep on declarative memory using visuospatial memory tasks have previously shown that cuing during SWS with an olfactory stimulus produced increases in fast spindle density over parietal regions [30,66]. Thus, in light of past studies and our present results, it is possible that the olfactory cue reactivated preferentially the spatial representation of the motor sequence during sleep, which in turn was enhanced via spindle activity in parietal regions.

### Spindle Frequency as a Mediator of the Effect of Olfactory Cuing on Performance Improvements

In the present paper, we show that the change in sleep spindle frequency over parietal regions mediated the relationship between cuing and offline gains. These results indicate that the experimental manipulation (i.e., cuing the memory of a motor task with an odor, or not) can predict both the level of offline gains as measured at retest and the increase in parietal spindle frequency before versus during stimulation. This mediation effect implies that, regardless of the presence of a conditioned stimulus or not, spindle frequency variations over the parietal region predict the level of gains in performance the next morning. Thus, the present findings provide the first evidence of a mediator effect of sleep spindles on the relationship between a TMR protocol and MSL offline gains. Taken together, the combination of the TMR experimental design used here, the specific increase in spindle characteristics yielded by cuing, and the mediating effect of spindle frequency on offline gains suggest that NREM2 sleep spindles are instrumental to sequential motor learning consolidation.

Again, it is difficult to explain why an increase in spindles of higher frequencies is particularly important for consolidating the memory trace associated with a newly acquired sequence of movements. As such changes were observed over Pz, the latter finding is in accord with the fact that sleep spindles detected over central and parietal regions are characterized by higher frequencies (called fast spindles) [67,68], and that these events have often been found to be associated with motor memory consolidation [15,18,34]. Furthermore, our results are consistent with those of innovative studies using combined functional magnetic resonance imaging (fMRI)/EEG recordings during sleep by Tyvaert and collaborators [69], who have shown a link between spindle activity and increased blood-oxygen-level dependent (BOLD) signal in the putamen, as well as others, who have reported that fast spindles are associated with increased activity in cortical motor regions and the hippocampus [70,71]—all structures that have been shown to play a significant role in motor learning.

## Conclusion

Although it is important to note that the present TMR study does not prove causality, as we did not manipulate directly the occurrence of sleep spindles, we believe that our results and the experimental design used here offer, to our knowledge, the first evidence toward an instrumental role of NREM2 sleep in the consolidation of motor sequence memory through the increased activity of sleep spindles over parietal regions. We show that cuing during NREM2 not only increases posterior spindle frequency, but also that these changes predict future performance on a motor sequence task. Based on the present findings, and those of other groups of investigators, we thus propose that NREM, and NREM2 sleep in particular, through the specific neuronal activity of sleep spindles, play a critical role in the consolidation process of a motor memory trace generated through practice of a new sequence of movements. More specifically, the increases in spindle amplitude, duration, and especially frequency observed during the stimulation phase (hence possibly facilitating the reactivation process) lead to a better consolidation process and ultimately to higher sequential motor performance the next day. However, further combined sleep/imaging studies are needed to investigate the neural correlates of the spindle-related reactivated memory trace from the enhancement through cuing of a conditioned stimulus.

## Method

### Participant Recruitment and Selection

#### Preselection

To be included in the study, eligible participants had to be right-handed, between 20 and 35 y old, and had to have no previous formal training playing a musical instrument nor any training as a professional typist, in order to control for pre-existing experience in tasks requiring highly coordinated finger movements. Obese individuals (BMI > 30), those using nicotine regularly, and users of recreational drugs were excluded. They also had to be free of any history of neurological, psychological, psychiatric, and sleep disorders. Furthermore, individuals who worked night shifts, were engaged in trans-meridian trips in the 3 mo prior to the study, or reported taking three or more servings of caffeinated beverages per day were not included in the study. All eligible participants had to have a score lower than ten on the Beck Anxiety Inventory [72] and the short version of the Beck Depression Inventory [73]. The quality of their sleep was assessed with the Pittsburgh Sleep Quality Index questionnaire [74].

#### Screening session

A total of 135 participants met the initial eligibility criteria prior to engaging in an overnight PSG screening (the main eligibility criterion prior to enrollment into the experimental night) in the sleep laboratory according to American Academy of Sleep Medicine guidelines [75]. PSG screening included EEG, electrooculography (EOG), leg and facial (submental) electromyography (EMG), thoracic and abdominal respiratory effort belts, and airflow; all of these measures were employed to identify signs of sleep disorders (e.g., insomnia, apnea, parasomnias, etc.), which were used as exclusion criteria. In addition, the screening night allowed us to objectively quantify the subjects’ sleep quality and to provide an opportunity for the participants to become acclimatized to the laboratory environment. The latter comprised sleep rooms that were built to be as comfortable as possible. Each of them was equipped with a single bed, comfortable mattress, nightstand, bookcase, lamp, and decorative plants. Although the rooms were windowless, curtains covering a portion of the wall were installed to give participants the feeling that the room had windows. The ceiling lights were controlled with a dimmer, and, importantly, air conditioning was centrally regulated through the hospital main system. Finally, sheets and beddings were changed every day and for each subject that participated in the study.

Upon arrival for the screening night, an olfactory threshold test was carried out to assess each individual’s level of scent detection (Sniffin’sticks, Burghart Medizintechnik, Germany). For selected participants, this olfactory threshold was used as a covariate measure for subsequent behavioral analyses, but did not constitute an exclusion criterion per se. Only participants without any signs of disordered sleep and who had a sleep efficiency over 80% were selected to participate in the study (PSG eligibility criterion) and were then invited to come back a week later for the experimental night. They were instructed to abstain from alcohol for the duration of the experiment. Subjects were asked to keep a strict sleep/wake schedule: go to bed between the hours of 10:00 p.m. and 1:00 am, wake up between 6:00 a.m. and 9:00 a.m., and abstain from taking naps during the day. To ensure that the participants adhered to this sleep/wake schedule during the week separating screening and experimentation nights, they were asked to wear an actigraph on their wrist (Actiwatch 2, Phillips Respironics). Complementary to the actigraph, participants were also asked to complete a sleep diary. Individuals who did not follow this strict sleep/wake schedule were not enrolled in the study and did not participate in the experimental night. Out of the 135 eligible individuals, eight were found to have at least one type of sleep disorder (e.g. bruxism, sleep apnea, periodic limb movement), 20 did not reach the 80% sleep efficiency threshold, six did not follow instructions regarding the sleep/wake cycles (following inspection of the actigraphy data), and nine voluntarily dropped out of the study.

#### Experimental session

Thus, after completing the screening night, a total of 92 participants were selected and enrolled in the study. Importantly, participants’ assignment into the experimental group took place after all eligibility criteria were applied and participants were screened out. Of the 92 enrolled participants, 18 were subsequently discarded from the analysis for the following reasons: seven were excluded due to low sleep efficiency (<75%) during the experimental night, three were due to technical problems (e.g., issue with the response box or the olfactometer), four were because they did not properly follow the instructions during the motor sequence task, and four were because their performance on the MSL task was considered as outlier. Thus, 76 participants were included in the behavioral analyses of the MSL task. They were distributed as follows: 25 subjects were included in the Cond-NREM2 group (mean age: 25.42 ± 4.4 y, 11 females), 23 in the Cond-REM group (mean age: 24.77 ± 4.2 y, nine females) and 28 in the NoCond group (mean age: 24.60 ± 4.9 y, 11 females). Of these, 12 participants were further discarded from the subsequent sleep and spindle analyses due to poorly recorded PSG data. Consequently, 64 participants were included in the sleep EEG analyses and were distributed as follows: 21 subjects were included in the Cond-NREM2 group (mean age: 25.5 ± 4.5 y, eight females), 21 in the Cond-REM group (mean age: 25.13 ± 4.2 y, nine females) and 22 in the NoCond group (mean age: 24.18 ± 4.4 y, ten females).

### Overall Experimental Design and Procedure

A week after the screening session, participants were invited again to the laboratory for the experimental night. Following proper installation of the EEG electrodes for polysomnographic recordings and the olfactometer apparatus, participants were randomly assigned to either of the three experimental groups (see next section and Fig 1B).

Prior to carrying out the MSL task, participants completed the Standford Sleepiness Scale (SSS) [76] to assess their subjective levels of sleepiness. Around 10:30 pm, they were then trained on the motor task, during which, depending on their experimental group, they were exposed or not to a rose-like odor through a nasal cannula. Overnight PSG recording began immediately following the training period. To increase sleep efficiency, exact timing for initiating the MSL training and for allowing subjects to sleep was adjusted according to each participant’s natural sleep onset preference. After 4 h of sleep recording time, participants were re-exposed to the rose-like odor during the subsequent episodes of the targeted sleep stage for a maximum of 60 min. To achieve maximum exposure time, several bouts of stimulation were necessary. Following the olfactory stimulation period, participants were allowed to complete their night of sleep until they reached 8 h of recording time. Participants were then retested on the SSS and MSL task 2 h after waking to reassess their performance after enough time to allow for sleep inertia to dissipate.

### Experimental Groups

Three groups of subjects participated in this study (see Fig 1B). The Cond-NREM2 and Cond-REM groups received the olfactory stimulus during the training session. This procedure allowed participants to establish a strong association between learning of the novel motor task and the rose-like smell. These two groups were then re-exposed for a maximum of 60 min to the same odor during either NREM2 or REM sleep in the second half of the night in order to determine the sleep stage during which optimization of the consolidation process takes place. One additional group, which was not exposed to the odor during the training session (even though a nasal cannula was in place), but was nevertheless exposed to the olfactory stimulus during NREM2 sleep, was included to control for the presence of odor during the encoding stage and the possible nonspecific effects of exposing subjects to an olfactory stimulation during NREM2 sleep (NoCond group). None of the subject was aware of his or her experimental group assignment, nor had any knowledge of these procedural differences.

### Motor Sequence Learning: Finger Sequence Task

Motor sequence learning was tested with an adapted version of the sequential finger tapping task first developed by Karni et al. [77]. Subjects were asked to practice an explicitly known eight-item sequence of finger movements (2-4-1-3-4-2-3-1, where 1 stands for the index finger and 4 for the little finger) using a procedure employed previously in the laboratory (e.g., [6]), except that subjects were asked to complete 24 blocks of practice of the sequence during the initial training session and eight others during the retest session. In order to verify that participants had explicitly memorized the motor sequence prior to training, participants were required to repeat the sequence until they were able to reproduce it three times in a row without error. During training, participants were required to practice the sequence by executing the finger movements as quickly as possible while making as few errors as possible, without looking at their hand. To do so, participants had to use a response box comprising four buttons, and press one button at a time with fingers of their left (non-dominant) hand. They had to practice the sequential movements for as long as a green cross (3 x 3 cm^2^) was displayed at the center of a computer screen. Unknown to subjects, the green cross was displayed until 80 finger movements were recorded (one block; ideally corresponding to the production of ten correct sequences). No information about the sequence or performance feedback was given to the participants during the task. During pre-sleep training, 24 blocks of motor sequence practice were interspersed with 30 s periods of rest, during which a red cross was displayed at the center of the screen. The retest session was exactly the same as the training session, but comprised only eight blocks of motor sequence practice and took place the following morning. The task was coded using the Cogent2000 toolbox (http://www.vislab.ucl.ac.uk/cogent.php) and implemented using MATLAB (Mathworks Inc., Sherbom, MA).

### Performance Assessment and Analyses of the GPI

Behavioral performance at a sequence motor learning task is often reported as speed (e.g., time between key presses, time for correct sequences, time per block) and/or accuracy (e.g., number of correct key presses per block, number of correct sequences). Most studies measured offline consolidation through change in speed, given that accuracy in this type of tasks is typically high and its fluctuations are minimal [64,78]. However, these small differences in accuracy at the individual level might reflect differences in motor strategy. For example, some participants might prioritize accuracy at the expense of speed while others might prefer to be faster even if it implies making more mistakes. Furthermore, the instructions provided to participants were explicitly to perform the sequence as quickly as possible while making as few errors as possible. Thus, in order to reflect these requirements and to account for individual strategy differences, we measured the subjects’ performance using a GPI.

Similar to indexes in previous research [79,80], the GPI was built based upon the following measures of speed and accuracy:

$$speed_{block}=\frac{t_{block}}{n}$$

$$accuracy_{block}=\frac{n-\;\sum_{block}correct}{n}$$

where *t* corresponds to the time in seconds to complete a block of training and *n* is the number of key presses within a block (80 in this experiment). Correct key presses were established using an algorithm identifying correct triplets ([2,4,1], [4,1,3], [1,3,4], [3,4,2], [4,2,3], [2,3,1], [3,1,2], [1,2,4]) in the original task’s sequence (2,4,1,3,4,2,3,1). The use of triplets and not pairs avoided the detection of false positive correct key presses. When a triplet that was not in the list was found, the last key press of this triplet was marked as incorrect.

In order to account for possible speed–accuracy trade-off, the following GPI was then computed:

$$GPI=\;e^{-speed}*e^{-accuracy}$$

where *e* is the mathematical constant, also known as the Euler’s number, and is defined as the base of the natural logarithm (~2.71828). The higher the GPI, the better the performance was (see Fig 2A). The GPI was used here as it takes into account the time taken to commit and recover from an error, which is typically longer than for correct key presses.

Based on performance of the MSL task during initial training (i.e., prior to group assignment), we identified participants who were outliers, as compared with the average performance of all participants. To do so, we first used a learning curve approach to describe the performance of each participant. Each subject’s GPI in the training session (24 blocks) was then fitted using this function:

$$f_{\left(y\right)}=\left(\left(S-A\right)*e^{\left(R*x\right)}\right)+A$$

Performance asymptote (*A*), starting point (*S*), slope (*R*), and adjusted r-squared were extracted from this fit—one value for each of these measurements per subject. On each of these measures, outliers were identified using the generalized extreme studentized deviate (ESD) [81]. The main advantage of the generalized-ESD method over other outlier tests is that it does not find a definite number of outliers, but only needs an upper bound for the possible number of outliers to be specified [82]. Since there was no a priori concerning the expected number of outliers, the upper bound was fixed to the total number of participants tested, excluding those who did not meet the required criteria (see Participant Recruitment and Selection section; *n* = 80). Finally, subjects who had two or more values extracted from the fitting curve that surpassed this threshold were considered as outliers and were rejected from further analyses.

### Olfactory Threshold, Stimulus Delivery, and Analyses

To minimize habituation to the odor, several precautions were taken. First, the olfactometer itself was kept in a separate location from the testing and sleeping rooms. Second, the manipulation of olfactory stimuli was always conducted outside the testing room. Finally, delivery of the stimulus was carried out using an ON/OFF block design procedure (see Fig 1D). During the ON blocks, the odor was sent for 1 s every 3 s. For the MSL training session, the ON blocks consisted of the period during which subjects were practicing the sequence, while the OFF blocks corresponded to the periods of 30 s of rest in-between. During the targeted stage of sleep, the odor was delivered on a 30 s ON/30 s OFF block design for a maximum of 60 min.

A solution of phenyl ethyl alcohol (PEA—concentration: 6.31 x 10^−3^ [% v/v]) and heavy mineral oil (solvent—USP/FCC) was used as the odorant source. PEA has a pleasant rose-like smell and is known as a pure odorant, that is, a chemical substance that stimulates the olfactory nerve exclusively, as opposed to a mixed olfactory/trigeminal odorant that could lead to unpleasant sensations (e.g. burning, itching, cooling, etc.) [83]. Importantly, several studies have shown that the presentation of an olfactory stimulus during sleep does not wake up subjects [84,85]. The 125 ml emulsion was stored in an air-tight 750 ml glass container connected to an olfactory delivery system using Teflon-coated tubes (Tygon SE-200, Saint-Gobain Performance Plastics) to deliver the odor to the subject via a Teflon-coated nasal cannula. Teflon does not easily bond to PEA molecules, thus maintaining a constant concentration of PEA in the airflow [86].

Although odorant concentrations did not vary between subjects, the length of exposure during sleep did vary due to inter-individual differences in sleep architecture. Therefore, analyses were conducted on both sets of participants (all subjects included in behavioral analyses [*n* = 76] and those included in PSG analyses [*n* = 64]) in order to verify that there was no significant difference in amount of stimulation between groups. One-way ANOVAs were performed on wake duration during cuing (within-subjects factor) and groups (between-subjects factor) to test for any differences between groups. We also investigated, with a similar analysis, the duration of cuing during REM sleep between groups. Finally, one-way ANOVAs were conducted on total exposure and duration of cuing during NREM2 sleep durations, within and between groups. Results from post-hoc univariate tests between Cond-NREM2 and NoCond groups were reported to verify that there was no difference.

The PEA concentration in the odorant solution was exactly the same for each participant. However, there were inter-individual differences in terms of their olfactory threshold. Each participant’s threshold was identified with the Sniffin’sticks test (Burghart Medizintechnik, Germany). A one-way ANOVA was thus performed to determine if there was a difference between groups (between-subjects factor) in olfactory threshold (within-subjects factor) as measured by this test.

### Analyses of Motor Sequence Learning and Consolidation

Learning during the evening training session was investigated using a mixed design ANOVA for repeated measures with blocks (*n* = 24) as the within-subjects factor and groups as the between-subjects factor. This analysis permitted us to ensure that all participants showed a learning effect during the evening session (main effect of block). It also assessed for differences in learning rate between groups (block x group interaction) and differences between groups in terms of overall MSL skill throughout the session (main effect of group).

In order to investigate the level of performance at the end of the training session (later used in the calculation for the level of consolidation), a mixed repeated-measures ANOVA was conducted, with the mean GPIs from the last four blocks of the evening MSL task as the repeated within-subjects factor and groups as the between-subjects factor. This analysis allowed us to determine whether all participants reached an asymptotic performance (main effect of block). It also provided information about the learning rate (block x group interaction) and level of performance (main effect of group) between groups.

The level of consolidation was assessed through a repeated-measures ANOVA conducted with the two sessions (training and retest) and eight blocks (i.e., the last four blocks of training and the first four blocks of the retest session) as repeated within-subjects factors and groups as the between-subjects factor. Four blocks used to calculate gains in performance were selected because it has been shown in several studies that there is a warm-up effect occurring during the first block of retest [87,88]. The physiological basis of the so-called warm-up effect are not well understood yet, but it is recognized that it tends to drastically inflate inter-key-press time during the first retest block. Thus, averaging four blocks reduced the inter-subject variance in performance and allowed for the inclusion of the first retest block. As it is highly possible that the duration of exposure to the olfactory stimulus and the olfaction threshold of each participant would have played an important role in the experimental manipulation occurring overnight, the latter analysis was carried out controlling for the subjects’ olfactory threshold (as measured with the Sniffin’sticks test) and the duration of exposure to the odor during sleep. Finally, as a post-hoc analysis, we conducted a one-way ANOVA on the difference in GPI between the morning and evening sessions between the three groups and carried out planned contrast analyses assessing the specific differences between groups.

Finally, the same behavioral analyses were also performed with participants who were only included in the EEG and spindle analyses in order to ensure that the results from the entire groups of participants did not differ from the sub-set who had good EEG data.

### PSG Recording

PSG recordings were acquired using a 16-channel, V-Amp 16 system (Brainamp, Brain Products GmbH, Gilching, Germany) from ten scalp derivations (F3, Fz, F4, C3, Cz, C4, P3, Pz, P4, Oz) referenced to linked mastoids (A1, A2). PSG signals were recorded continuously (at <5K Ohm) during the whole night using Recorder software (Brain Products) and were visually inspected online for quality. Signals were digitalized at 250 samples per second (high pass filter = 0.3 Hz, low pass filter = 70 Hz). PSG measurements included EEG, electro-oculogram (EOG), and bipolar submental electromyogram (EMG) electrodes, as well as a nasal airflow thermistor (Braebon, Ottawa, Canada) to monitor respiratory effort.

For all PSG recordings, including online scoring and stimulation periods, sleep stages were visually identified in 30 s epochs displaying EEG (high pass filter = 0.3 Hz, low pass filter = 35 Hz) from central and occipital derivations (C3, C4, and Oz) referenced to average mastoids (A1 and A2), EOG (high pass filter = 0.3 Hz, low pass filter = 35 Hz) from the lateral outer canthus of each eye, and bipolar submental EMG (high pass filter of 10 Hz). Periods of cortical arousal or movement during sleep were identified using an automated detector when movement continuously exceeded 100 μV for more than 100 ms.

### EEG Preprocessing

#### Sleep architecture

PSG recordings were sleep stage scored according to standard criteria [89] using 30 second epochs. Analysis of the sleep architecture was conducted on distinct parts of the night, depending on the timing of the stimulus administration and according to the experimental protocol (see Fig 1C). More specifically, the sleep period occurring before onset of the olfactory stimulation was defined as the pre-stimulation phase, while sleep following the beginning of the stimulation phase was defined as the from-stimulation phase. Finally, the period of sleep defined as during-stimulation comprised only the periods of stimulation in the targeted sleep stage (e.g., NREM2 or REM).

#### Sleep spindle detection and channel localization

Sleep spindles were automatically detected from Fz, Cz, and Pz in non-REM sleep using Brain Products (Brain Products GmbH, Gilching, Germany) Analyzer software (Version 2.1) with a method described by Ray et al. [41]. The automated spindle detection technique used a complex demodulation transformation [90] to extract the power of each data point between 11 Hz and 17 Hz and is similar to the root mean square method employed to transform raw EEG signal [91]. Peak amplitude (max peak-to-peak value, in μV), duration (offset–onset, in seconds), and peak frequency (max peak-to-peak distance, in Hz) were calculated from the original EEG signal filtered from 11 Hz to 16 Hz. Peak frequency and amplitude were extracted, for each individual sleep spindle, using fast Fourier transforms, as indicated by the power spectrogram. This represents the frequency and amplitude that had the greatest power for each spindle. Spindle density was also calculated based upon the number of spindles per minute. Measures of peak amplitude, duration, and peak frequency for each spindle were extracted from all three sites (Fz, Cz, Pz) for analyses.

Given that the same spindle could be detected more or less simultaneously on multiple electrodes as a result of co-detection [92] or propagation [54,67,93], and in line with published and recommended methods [54,94], we sought to separate them by their principal recording sites (i.e., Fz, Cz, or Pz) as a data preprocessing stage. To do so, we used the onset of each spindle as a marker to determine their primary localisation. For each detected spindle, time-lapse windows were created on the two other derivations before and after a spindle onset (200 ms for adjacent sites, e.g., Pz and Cz; 400 ms for nonadjacent sites, Fz and Pz). Spindles were systematically categorized into two groups. First, as pure spindles—that is, when an event (a spindle) occurred in only one derivation within the given time window. Second, multi-site spindles were events with occurrence on at least one other electrode within the time frame. Taken together, these spindles were considered as a single event detectable from other recording sites and formed multi-site groups of spindles. Spindles from the latter class were again divided into two subcategories: source or rejected. Source spindle were defined by their onset as the first ones to occur in a multi-site group and were categorized by recording sites. Finally, the rejected spindles were multi-site spindles that occurred on other derivations after the source spindle and weren’t included in the analyses. Importantly, although some recorded events were rejected, no spindle was totally discarded per se, as source spindles were kept for analyses. The identification and classification of spindles was carried out using software coded in MATLAB (Mathworks Inc., Sherbom, MA).

Several studies have previously utilized a fast/slow spindle classification in order to investigate the function of spindles. Among others, techniques using individual threshold [95] have been described. In contrast to the latter approach, the present technique is based solely on the spindle’s derivation. This method was used, in part, because it reduces the risk of losing information from slower spindles occurring in parietal regions and faster spindles in frontal regions. To observe changes produced based upon the categorization on sleep spindle distributions, we reported the percentage of rejected spindles originally detected on Fz, Cz, and Pz sites, and then analyzed with paired-sample *t* tests the spindle frequency median changes of the distribution caused by the filtering on Fz, Cz, and Pz. Furthermore, we investigated the categorization algorithm effect on Pz spindle frequency, by comparing the median and mean, in pre-matched and during-stimulation sleep periods (see S1 Text).

### EEG Analyses

#### Sleep spindle analyses and stimulation period

Statistical comparisons of spindles were carried out on Fz, Cz, and Pz derivations. For each derivation, peak amplitude, duration, peak frequency, and density of sleep spindles were analysed. Given that stimulation during sleep was carried out toward the end of the night and that most participants had little or no SWS at the beginning of the re-exposure period, spindle analyses were conducted using NREM2 sleep only.

Furthermore, in order to be able to compare between periods with and without stimulation, a period of sleep of the same length as during-stimulation was selected from the pre-stimulation period for each subject. Starting from the last epoch of NREM2 and going backward in the recording of the pre-stimulation period, the same number of epochs as in the during-stimulation period was selected for each subject. This period was defined as pre-matched. It served as a baseline and permitted us to compare the events during the stimulation closest to the previous periods of NREM2 sleep (Fig 1C).

Given that one of our main hypotheses was that cuing during NREM2 sleep would induce changes in sleep spindles, we used one-way ANOVAs to assess the differences in spindle characteristics (within-subjects factor) between groups (between-subjects factor), first in the pre-matched period to ensure that all groups were similar before cuing, and second in the during-stimulation sleep periods. We also investigated the effect of cuing by measuring and analyzing difference (Δ) in spindle characteristics between two sleep periods: pre-matched (baseline) versus during-stimulation (cuing). This differential score was calculated by measuring the percent change for each spindle characteristic between the two periods of interest. One-way ANOVA analyses were used to investigate the pre-matched versus during-stimulation differences (within-subjects factor) between the Cond-NREM2 and NoCond groups (between-subjects factor). We also assessed the nature of changes in spindle frequency probed by the stimulation by analyzing changes in specific bins of 0.5 Hz (11–17 Hz) between the pre-matched and during-stimulation periods for both the Cond-NREM2 and NoCond groups with Chi^2^ analyses (Bonferroni-corrected for the number of bins). This analysis provided information about the specific ranges of frequency that were modified by the manipulation.

In order to provide a better and unbiased estimation of the group differences regarding various spindle characteristics (frequency, amplitude, duration, density), we performed a bootstrap analysis [96]. Specifically, for each spindle characteristic (e.g., frequency) and for each group, we generated 5,000 data samples (sampling with replacement), equal in size with the original sample in both groups (*n* = 21 for Cond-NREM2 and *n* = 22 for NoCond; see S1 Text for more details about this procedure). To control for multiple independent comparisons, a Bonferroni correction for 12 comparisons (*p* < 0.004) was applied on the bootstrap results (3 electrodes x 4 spindle characteristics).

#### Mediation analyses

Finally, we conducted mediation analyses, using change between sleep periods in spindle characteristics as a mediator, experimental conditions as an independent variable, and gains in performance as the outcome measure, using PROCESS, an SPSS add-on module [97]. We used a single mediator model, and the mediation effect was tested using a bias corrected and accelerated bootstrap procedure (1,000 samples—[BCa]) [98].

### Ethics Statement

The present study was revised and approved by an institutional ethics committee (“Comité mixte d’éthique de la recherche du Regroupement Neuroimagerie/Québec;” ID: CMER-RNQ 09-10-026). Upon their arrival at the sleep laboratory for the screening night, all participants were asked to read carefully and sign the written consent form.

## Supporting Information

S1 Fig Duration of exposure to the olfactory stimulus during sleep stages. The great majority of the olfactory stimulation occurred during the targeted stage in each group (Cond-NREM2 and NoCond: NREM2 sleep; Cond-REM: REM). No differences were found when assessing the total exposure time or targeted durations between the three groups. Also, no difference was found when looking at the duration of exposure during NREM2 sleep between the Cond-NREM2 and No-Cond groups. Data deposited in the Dryad repository: http://dx.doi.org/10.5061/dryad.b4t60 [40].
(TIF)

S2 Fig MSL task: speed and accuracy. **(A) MSL inter-key presses speed at training and retest.** Speed was computed using the average inter-key presses time per block for each subject. Each curve represents the mean for a group, and each point consists of a single block of training or retest. Standard error values are represented by error bars. Repeated measures ANOVA on inter-key press time of the last four blocks of training and four first blocks of retest revealed a main effect of session (F1, 71 = 12.126, *p* = .001) and a session x group interaction (F2, 71 = 5.367, *p* = .007), demonstrating that, while all participants showed gains in performance between the two sessions, there was a significant group difference in the level of motor skill consolidation. Planned contrasts analyses revealed that the Cond-NREM2 group exhibited significantly higher gains in performance than the NoCond (*p* = .002) group and was close to significance compared to the Cond-REM group (*p* = .06). The results of the Cond-REM and NoCond groups did not differ significantly (p = .21). **(B) MSL number of errors per block.** Each point represents the average number of key press errors made during a block of practice. As for speed, each point is a block of MSL training or retest, groups are identified by different shapes and colors, and error bars consist of standard error values.
(TIF)

S3 Fig Sorted bootstrap random sampling changes in sleep spindle amplitude at Pz. 5,000 random change in amplitude samples from Cond-NREM2 and NoCond groups were extracted and sorted in ascending and descending order, respectively. This procedure allowed us to compare the highest value from the NoCond group with the smallest value of the Cond-NREM2. The sorted value lines crossed each other at index #6 (NoCond>Cond-NREM2 in six; Cond-NREM2>NoCond in 4,994 cases out of 5,000; see inlet). This analysis yielded a significant difference between Cond-NREM2 and NoCond groups (*p* = .0012). Data deposited in the Dryad repository: http://dx.doi.org/10.5061/dryad.b4t60 [40].
(TIF)

S4 Fig Sorted bootstrap random sampling changes in sleep spindle frequency at Pz. 5,000 random change in frequency samples from Cond-NREM2 and NoCond groups were extracted and sorted in ascending and descending order, respectively. As with amplitude, this procedure allowed us to compare the highest value from the NoCond group with the smallest value of the Cond-NREM2 group. The sorted value lines crossed each other at index #13 (NoCond>Cond-NREM2 in 13; Cond-NREM2>NoCond in 4,987 cases out of 5,000; see inlet). This analysis yielded a significant difference between Cond-NREM2 and NoCond groups (*p* = .0026). doi:10.5061/dryad.b4t60.
(TIF)

S1 Table Olfactory stimulation during sleep. Duration is shown in minutes (S.E.: standard error). Analyses were carried out separately using data from all participants included in the behavioral analyses or from the sub-set of subjects included in the PSG analyses. The two sets of data yielded a similar pattern of results. One-way ANOVAs were performed to assess whether there were any group differences in wake, sleep stages, or total duration of stimulation. Importantly, the results did not reveal any difference in length of either wake or total exposure time between groups, but yielded the expected differences in exposure time between groups during the two targeted sleep stages (i.e., NREM2 and REM). Furthermore, post-hoc univariate tests on the duration of cuing during NREM2 sleep and total exposure duration between the Cond-NREM2 and NoCond groups did not reveal significant differences, showing that both groups had received a similar amount of odor during the stimulation period.
(DOCX)

S2 Table Sleep architecture. All sleep measurements are presented in minutes, except for sleep efficiency, which corresponds to a percentage calculated from the ratio of TST on TRT. Standard errors are reported (S.E.). One-way ANOVAS were conducted for each sleep characteristic to determine whether there were any significant differences in sleep architecture between groups before, as well as from the onset of, the cuing period. As expected, there were no significant group differences in any of the sleep phases and characteristics.
(DOCX)

S3 Table Spindles characteristics for each sleep period during NREM2 at Pz. Amplitude is reported in μV, frequency is in Hz, spindle density is in number of spindles per minute of sleep, and duration is in seconds. Standard errors are reported (S.E.). Independent-sample *t* tests were conducted on the mean of each spindle characteristic to test differences between groups. *t* tests were also performed on Cond-NREM2 and NoCond groups for the sleep periods before and from the onset of exposure to the odor cue. It is important to note that no significant differences were found between groups in sleep spindles for any of the sleep periods.
(DOCX)

S1 Text Additional supporting materials, methods, and results sections. (DOCX)

## Abbreviations

BOLD: blood-oxygen-level dependent
EEG: electroencephalography
ESD: extreme studentized deviate
fMRI: functional magnetic resonance imaging
GPI: global performance index
MSL: motor sequence learning
NREM: non-REM sleep
NREM2: stage 2 non-REM sleep
PSG: polysomnographic
REM: rapid eye movement
SE: sleep efficiency
SRTT: serial reaction-time test
SWS: slow wave sleep
TMR: targeted memory reactivation
TRT: total recording time
TST: total sleep time
