# Supplementary figures and images for: NREM2 and Sleep Spindles Are Instrumental to the Consolidation of Motor Sequence Memories
Source: PLoS Biol. 2016 Mar 31;14(3):e1002429. doi: 10.1371/journal.pbio.1002429 (PMC4816304; doi:10.1371/journal.pbio.1002429)

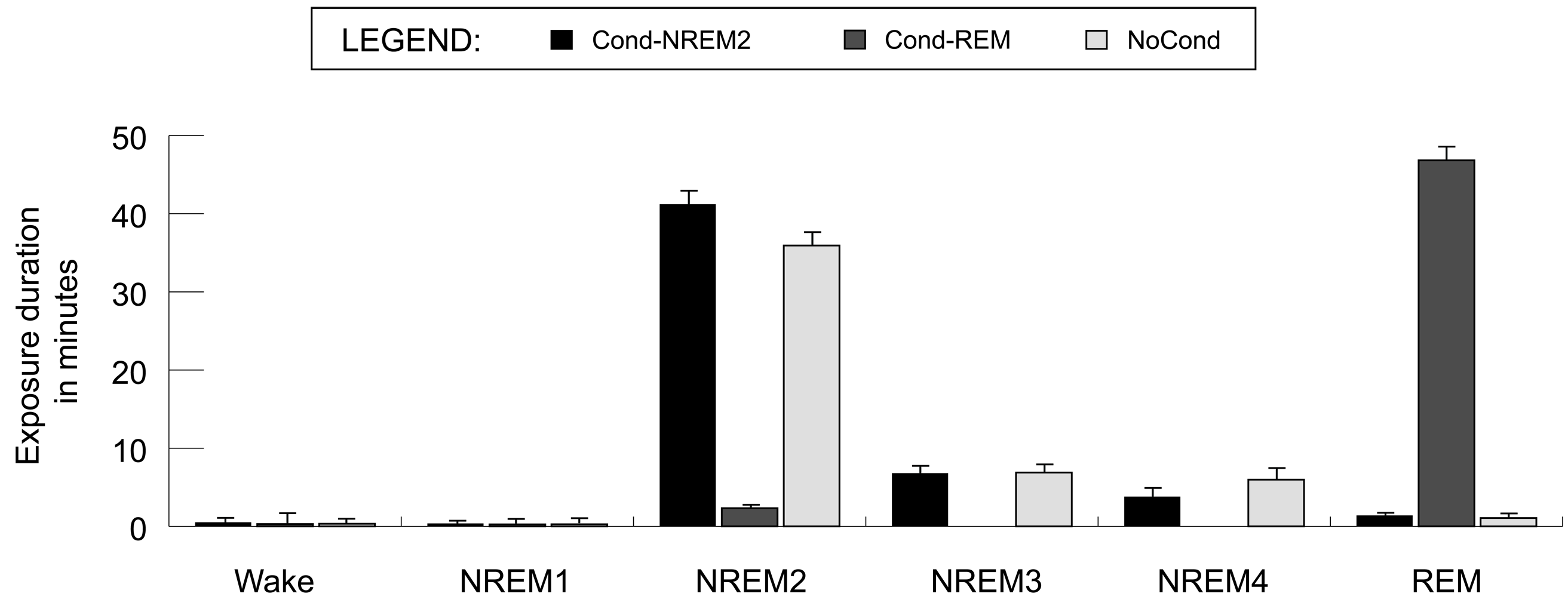

Supplement: S1 Fig — The great majority of the olfactory stimulation occurred during the targeted stage in each group (Cond-NREM2 and NoCond: NREM2 sleep; Cond-REM: REM). No differences were found when assessing the total exposure time or targeted durations between the three groups. Also, no difference was found when looking at the duration of exposure during NREM2 sleep between the Cond-NREM2 and No-Cond groups. Data deposited in the Dryad repository: http://dx.doi.org/10.5061/dryad.b4t60 [40]. (TIF) [file pbio.1002429.s001.tif]

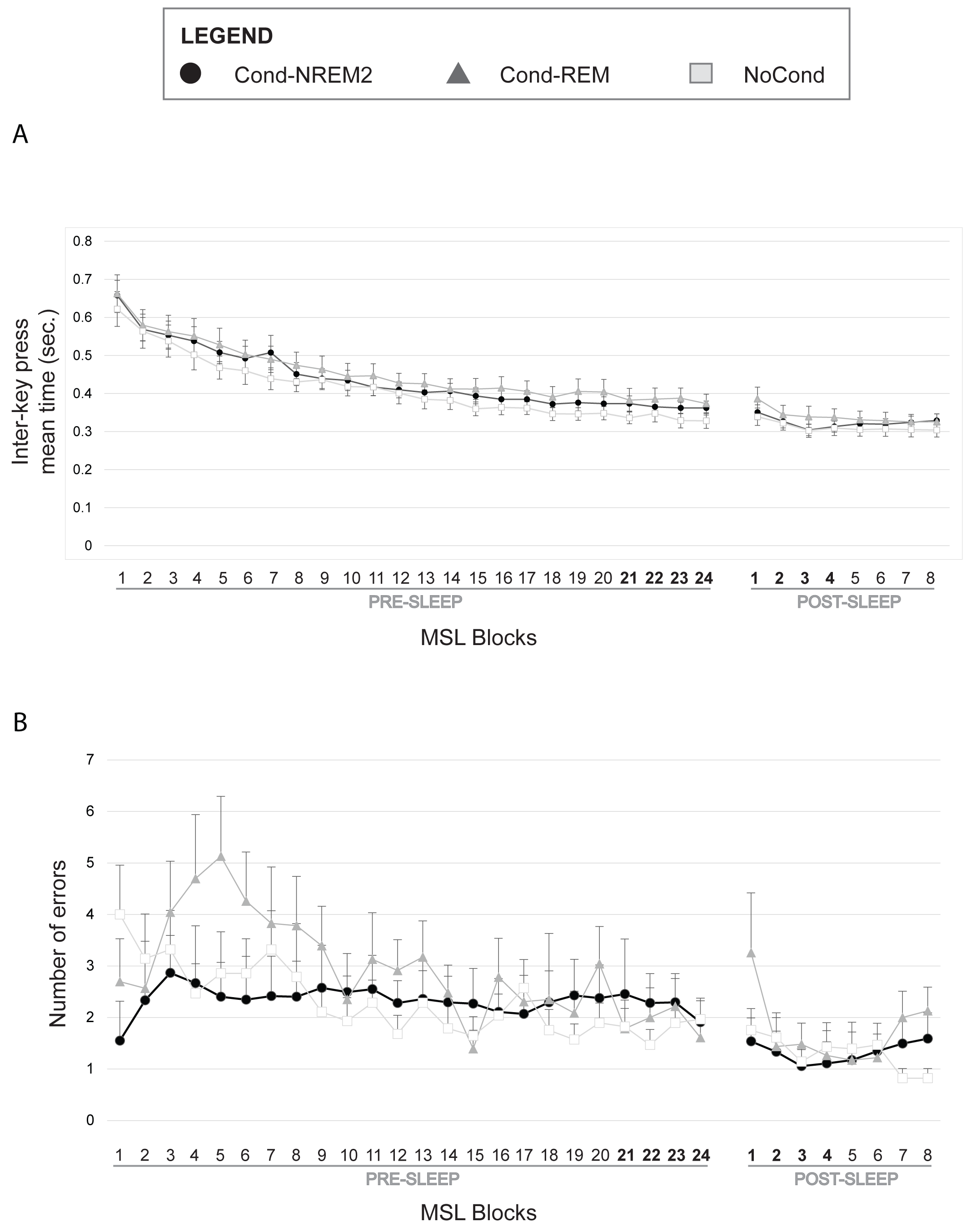

Supplement: S2 Fig — (A) MSL inter-key presses speed at training and retest. Speed was computed using the average inter-key presses time per block for each subject. Each curve represents the mean for a group, and each point consists of a single block of training or retest. Standard error values are represented by error bars. Repeated measures ANOVA on inter-key press time of the last four blocks of training and four first blocks of retest revealed a main effect of session (F1, 71 = 12.126, p = .001) and a session x group interaction (F2, 71 = 5.367, p = .007), demonstrating that, while all participants showed gains in performance between the two sessions, there was a significant group difference in the level of motor skill consolidation. Planned contrasts analyses revealed that the Cond-NREM2 group exhibited significantly higher gains in performance than the NoCond (p = .002) group and was close to significance compared to the Cond-REM group (p = .06). The results of the Cond-REM and NoCond groups did not differ significantly (p = .21). (B) MSL number of errors per block. Each point represents the average number of key press errors made during a block of practice. As for speed, each point is a block of MSL training or retest, groups are identified by different shapes and colors, and error bars consist of standard error values. (TIF) [file pbio.1002429.s002.tif]

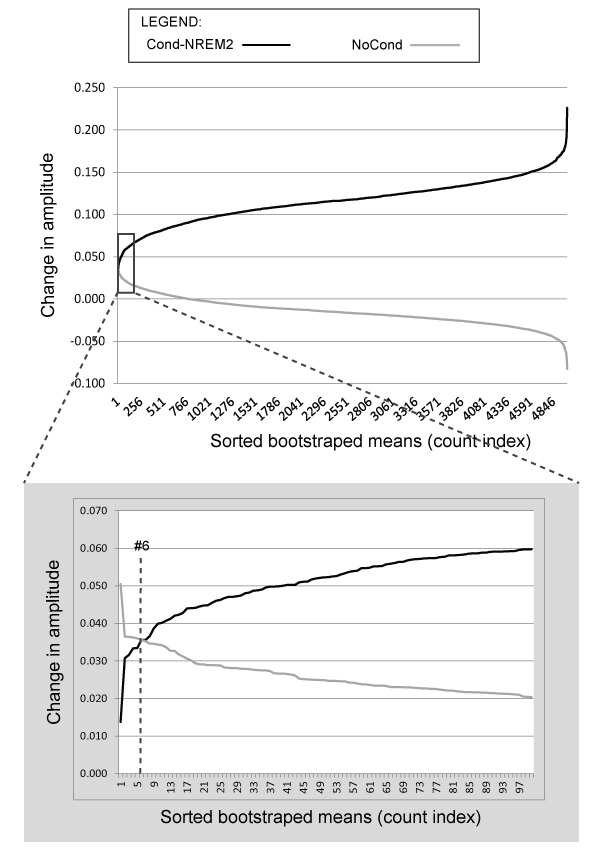

Supplement: S3 Fig — 5,000 random change in amplitude samples from Cond-NREM2 and NoCond groups were extracted and sorted in ascending and descending order, respectively. This procedure allowed us to compare the highest value from the NoCond group with the smallest value of the Cond-NREM2. The sorted value lines crossed each other at index #6 (NoCond>Cond-NREM2 in six; Cond-NREM2>NoCond in 4,994 cases out of 5,000; see inlet). This analysis yielded a significant difference between Cond-NREM2 and NoCond groups (p = .0012). Data deposited in the Dryad repository: http://dx.doi.org/10.5061/dryad.b4t60 [40]. (TIF) [file pbio.1002429.s003.tif]

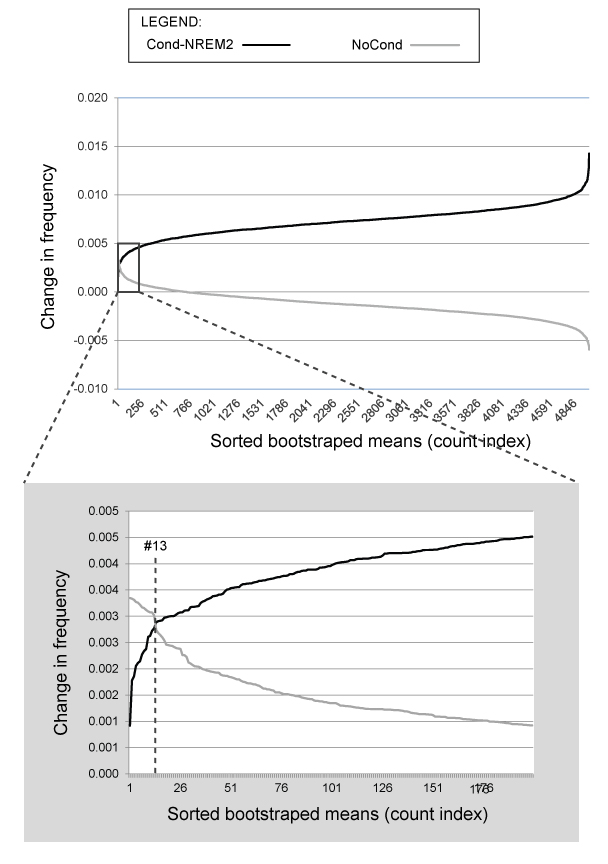

Supplement: S4 Fig — 5,000 random change in frequency samples from Cond-NREM2 and NoCond groups were extracted and sorted in ascending and descending order, respectively. As with amplitude, this procedure allowed us to compare the highest value from the NoCond group with the smallest value of the Cond-NREM2 group. The sorted value lines crossed each other at index #13 (NoCond>Cond-NREM2 in 13; Cond-NREM2>NoCond in 4,987 cases out of 5,000; see inlet). This analysis yielded a significant difference between Cond-NREM2 and NoCond groups (p = .0026). doi:10.5061/dryad.b4t60. (TIF) [file pbio.1002429.s004.tif]
