# Supplementary material for: NREM2 and Sleep Spindles Are Instrumental to the Consolidation of Motor Sequence Memories
Source: PLoS Biol. 2016 Mar 31;14(3):e1002429. doi: 10.1371/journal.pbio.1002429 (PMC4816304; doi:10.1371/journal.pbio.1002429)
Supplement: S1 Table — Duration is shown in minutes (S.E.: standard error). Analyses were carried out separately using data from all participants included in the behavioral analyses or from the sub-set of subjects included in the PSG analyses. The two sets of data yielded a similar pattern of results. One-way ANOVAs were performed to assess whether there were any group differences in wake, sleep stages, or total duration of stimulation. Importantly, the results did not reveal any difference in length of either wake or total exposure time between groups, but yielded the expected differences in exposure time between groups during the two targeted sleep stages (i.e., NREM2 and REM). Furthermore, post-hoc univariate tests on the duration of cuing during NREM2 sleep and total exposure duration between the Cond-NREM2 and NoCond groups did not reveal significant differences, showing that both groups had received a similar amount of odor during the stimulation period. (DOCX) [file pbio.1002429.s005.docx]

**Table S1. Olfactory stimulation during sleep.**

|  | **Cond-NREM2** | | **Cond-REM** | | **NoCond** | | **F** | **p** | ***(Cond-NREM2 vs NoCond)*** |
| --- | --- | --- | --- | --- | --- | --- | --- | --- | --- |
|  |  |  |  |  |  |  |  |  | p |
| ***All subjects*** | Mean | S.E. | Mean | S.E. | Mean | S.E. | **F*_(2,73)_*** |  |  |
| Wake | 0.5 | 0.1 | 0.3 | 0.1 | 0.3 | 0.1 | 0.449 | 0.64 | 1.00 |
| NREM1 | 0.3 | 0.1 | 0.4 | 0.1 | 0.2 | 0.1 | 1.206 | 0.30 | 0.74 |
| NREM2 | 39.0 | 2.2 | 2.7 | 2.3 | 32.0 | 2.1 | 73.127 | < 0.01 | 0.07 |
| SWS | 12.0 | 1.8 | < 0.1 | 1.9 | 14.6 | 1.7 | 18.446 | < 0.01 | 0.89 |
| REM | 1.4 | 1.6 | 44.8 | 1.7 | 1.9 | 1.5 | 230.441 | < 0.01 | 1.00 |
| Total exposure | 53.4 | 1.6 | 48.4 | 1.7 | 49.2 | 1.5 | 2.671 | 0.08 | 0.19 |
|  |  |  |  |  |  |  |  |  |  |
| ***Only subjects in PSG analyses*** | Mean | S.E. | Mean | S.E. | Mean | S.E. | **F*_(2,61)_*** |  |  |
| Wake | 0.5 | 0.2 | 0.3 | 0.2 | 0.3 | 0.2 | 0.355 | 0.70 | 1.00 |
| NREM1 | 0.4 | 0.1 | 0.3 | 0.1 | 0.2 | 0.1 | 0.614 | 0.54 | 0.84 |
| NREM2 | 40.9 | 2.7 | 2.7 | 2.3 | 35.6 | 2.2 | 83.794 | < 0.01 | 0.30 |
| SWS | 10.7 | 1.9 | 0 | 0 | 12.8 | 1.9 | 12.764 | < 0.01 | 1.00 |
| REM | 1.6 | 1.5 | 46.4 | 1.5 | 1.2 | 1.5 | 291.780 | < 0.01 | 1.00 |
| Total exposure | 54.1 | 1.7 | 50.0 | 1.7 | 50.2 | 1.6 | 2.001 | 0.14 | 0.27 |

Legend: Duration is shown in minutes (S.E.: standard error). Analyses were carried out separately using data from all participants included in the behavioral analyses or from the sub-set of subjects included in the PSG analyses. The two sets of data yielded a similar pattern of results. One-way ANOVAs were performed to assess whether there were any group differences in wake, sleep stages or total duration of stimulation. Importantly, the results did not reveal any difference in length of either wake or total exposure time between groups, but yielded the expected differences in exposure time between groups during the two targeted sleep stages (i.e. NREM2 and REM). Furthermore, post-hoc univariate tests on the duration of cuing during NREM2 sleep and total exposure duration between the Cond-NREM2 and NoCond groups did not reveal significant differences, showing that both groups had received a similar amount of odor during the stimulation period.
