# Supplementary material for: NREM2 and Sleep Spindles Are Instrumental to the Consolidation of Motor Sequence Memories
Source: PLoS Biol. 2016 Mar 31;14(3):e1002429. doi: 10.1371/journal.pbio.1002429 (PMC4816304; doi:10.1371/journal.pbio.1002429)
Supplement: S2 Table — All sleep measurements are presented in minutes, except for sleep efficiency, which corresponds to a percentage calculated from the ratio of TST on TRT. Standard errors are reported (S.E.). One-way ANOVAS were conducted for each sleep characteristic to determine whether there were any significant differences in sleep architecture between groups before, as well as from the onset of, the cuing period. As expected, there were no significant group differences in any of the sleep phases and characteristics. (DOCX) [file pbio.1002429.s006.docx]

**Table S2. Sleep architecture.**

|  | **Cond-NREM2** | | **Cond-REM** | | **NoCond** | | **F_(2,61)_** | **p** | |
| --- | --- | --- | --- | --- | --- | --- | --- | --- | --- |
|  |  |  |  |  |  |  |  |  | |
| ***Pre-stimulation*** | Mean | S.E. | Mean | S.E. | Mean | S.E. |  |  | |
| Wake | 27.1 | 5.0 | 28.8 | 4.5 | 34.8 | 6.7 | 0.531 | 0.59 | |
| NREM1 | 10.1 | 1.2 | 12.0 | 2.0 | 12.0 | 1.8 | 0.416 | 0.66 | |
| NREM2 | 116.9 | 9.0 | 113.7 | 10.1 | 108.9 | 6.9 | 0.217 | 0.81 | |
| SWS | 97.2 | 5.3 | 101.5 | 8.4 | 84.1 | 7.4 | 1.635 | 0.20 | |
| REM | 44.3 | 4.3 | 40.7 | 2.8 | 38.7 | 4.2 | 0.564 | 0.57 | |
| Movement | 0.1 | <0.1 | 0.1 | 0.1 | 0.3 | 0.2 | 1.356 | 0.27 | |
| Total Recording Time (TRT) | 297.5 | 12.0 | 299.1 | 8.6 | 280.1 | 9.1 | 1.045 | 0.36 | |
| Total Sleep Time (TST) | 268.6 | 10.8 | 267.9 | 6.0 | 243.8 | 9.0 | 2.584 | 0.08 | |
| Sleep Efficiency (%) | 91% | <0.1 | 90% | <0.1 | 87% | <0.1 | 1.076 | 0.35 | |
|  |  |  |  |  |  |  |  |  | |
| ***From-stimulation*** |  |  |  |  |  |  |  |  | |
| Wake | 22.4 | 4.4 | 22.0 | 4.6 | 17.3 | 2.4 | 0.558 | 0.58 | |
| NREM1 | 7.6 | 1.3 | 7.3 | 1.0 | 8.0 | 0.8 | 0.109 | 0.90 | |
| NREM2 | 82.9 | 4.8 | 77.9 | 5.8 | 85.7 | 5.2 | 0.567 | 0.57 | |
| SWS | 25.4 | 4.4 | 25.5 | 5.7 | 28.7 | 4.3 | 0.151 | 0.86 | |
| REM | 48.2 | 4.8 | 62.9 | 4.6 | 57.4 | 4.7 | 2.442 | 0.10 | |
| Movement | 0.1 | 0.1 | 0.0 | 0.0 | 0.3 | 0.2 | 1.239 | 0.30 | |
| Total Recording Time (TRT) | 187.6 | 10.9 | 197.3 | 6.7 | 199.4 | 8.1 | 0.515 | 0.60 | |
| Total Sleep Time (TST) | 164.1 | 10.3 | 173.5 | 8.4 | 179.8 | 8.5 | 0.760 | 0.47 | |
| Sleep Efficiency (%) | 87% | <0.1 | 86% | <0.1 | 90% | <0.1 | 0.514 | 0.60 |  |

Legend: All sleep measurements are presented in minutes, except for sleep efficiency which corresponds to a percentage calculated from the ratio of TST on TRT. Standard errors are reported (S.E.). One-way ANOVAS were conducted for each sleep characteristic to determine whether there were any significant differences in sleep architecture between groups before, as well as from the onset of the cuing period. As expected, there were no significant group differences in any of the sleep phases and characteristics.
