# Supplementary material for: NREM2 and Sleep Spindles Are Instrumental to the Consolidation of Motor Sequence Memories
Source: PLoS Biol. 2016 Mar 31;14(3):e1002429. doi: 10.1371/journal.pbio.1002429 (PMC4816304; doi:10.1371/journal.pbio.1002429)
Supplement: S3 Table — Amplitude is reported in μV, frequency is in Hz, spindle density is in number of spindles per minute of sleep, and duration is in seconds. Standard errors are reported (S.E.). Independent-sample t tests were conducted on the mean of each spindle characteristic to test differences between groups. t tests were also performed on Cond-NREM2 and NoCond groups for the sleep periods before and from the onset of exposure to the odor cue. It is important to note that no significant differences were found between groups in sleep spindles for any of the sleep periods. (DOCX) [file pbio.1002429.s007.docx]

**Table S3. Spindles characteristics for each sleep period during NREM2 at Pz.**

|  | **Cond-NREM2** | | **NoCond** | |  |  |
| --- | --- | --- | --- | --- | --- | --- |
|  | m (n=21) | | m (n=22) | | t_41_ | p |
|  |  |  |  |  |  |  |
| ***Pre-matched*** | Mean | S.E. | Mean | S.E. |  |  |
| Amplitude | 67.58 | 9.45 | 73.00 | 10.60 | -0.380 | .71 |
| Frequency | 13.33 | 0.11 | 13.34 | 0.11 | -0.082 | .94 |
| Density | 3.47 | 0.12 | 3.64 | 0.15 | -0.892 | .38 |
| Duration | 0.58 | 0.02 | 0.57 | 0.03 | 0.145 | .89 |
|  |  |  |  |  |  |  |
| ***During-stimulation*** |  |  |  |  |  |  |
| Amplitude | 71.98 | 9.25 | 69.63 | 11.26 | 0.160 | .87 |
| Frequency | 13.42 | 0.11 | 13.27 | 0.12 | 0.890 | .38 |
| Density | 3.48 | 0.13 | 3.80 | 0.13 | -1.630 | .11 |
| Duration | 0.63 | 0.04 | 0.60 | 0.04 | 0.659 | .51 |

Legend: Amplitude is reported in µV, frequency is in Hz, spindle density is in number of spindles per minute of sleep and duration is in second. Standard errors are reported (S.E.). Independent-samples t-tests were conducted on the mean of each spindle characteristic to test differences between groups. T-tests were also performed on Cond-NREM2 and NoCond groups for the sleep periods before and from the onset of exposure to the odor cue. It is important to note that no significant differences were found between groups in sleep spindle for any of the sleep period.
