# Supplementary material for: NREM2 and Sleep Spindles Are Instrumental to the Consolidation of Motor Sequence Memories
Source: PLoS Biol. 2016 Mar 31;14(3):e1002429. doi: 10.1371/journal.pbio.1002429 (PMC4816304; doi:10.1371/journal.pbio.1002429)
Supplement: S1 Text — (DOCX) [file pbio.1002429.s008.docx]

**PLOS Biology**

**Supporting Information**

**NREM2 and Sleep Spindles are Instrumental to the Consolidation of Motor Sequence Memories**

**Samuel Laventure, Stuart Fogel, Ovidiu Lungu, Geneviève Albouy, Pénélope Sévigny-Dupont, Catherine Vien, Chadi Sayour, Julie Carrier, Habib Benali, Julien Doyon**

**Supporting Information**

1. Supporting Experimental Procedures
2. Supporting Results
3. Supporting References

**1. Supporting Experimental Procedure**

**Changes in sleep spindle characteristics bootstrap and control for multiple comparisons**

In order to provide a better and unbiased estimation of the group differences regarding various spindle characteristics (frequency, amplitude, duration, density) we performed a bootstrap analysis (1). Specifically, for each spindle characteristic (ex. frequency) and for each group we generated 5000 data samples, equal in size with the original sample in each group (N=21 for Cond-NREM2 and N=22 for NoCond). These samples were drawn at random, with replacement, from their respective original samples. For each of them, the mean of the spindle characteristic was computed, thus yielding a distribution of 5000 means for each spindle characteristic and group. In order to avoid any ties, we calculated each mean with a precision of six digits. Given that the overall mean of Cond-NREM2 was higher than that of NoCond, we sorted the 5000 means of Cond-NREM2 in ascending order, and the 5000 means of NoCond in descending order and we paired them. Then, we computed the group difference between each of these sorted mean pairs. This procedure allowed us to compare the highest value from NoCond with the smallest value of Cond-NREM2, the second-highest value from NoCond with the second-smallest value of Cond-NREM2, etc. Given that we expected a difference in favor of Cond-NREM2 (experimental hypothesis), the null hypothesis is reflected in the opposite situation, whenever NoCond>Cond-NREM2. Thus, following the described procedure, one can calculate the probability for the Type I error by counting in how many cases the mean of NoCond is greater than that of Cond-NREM2, out of the 5000 mean pairs. For example, if in 100 out of 5000 pairs, the NoCond> Cond-NREM2, then the probability of Type I error when we claim the opposite, that Cond-NREM2 mean is greater than NoCond mean, is 100/5000=0.02.

**2. Supporting Results**

**Additional behavioral analyses**

Additional behavioral analyses were carried out, including the data from subjects considered as outliers. Accordingly, a repeated measure ANOVA was first conducted on data from the last 4 blocks of the training session and the first 4 blocks of the retest session. Similarly to the results reported in the manuscript, this new analysis revealed a main effect of session (F_1, 75_ = 10.033, p = .002) and a session x group interaction (F_2, 75_ = 3.997, p = .022), demonstrating that while all participants showed gains in performance between the two sessions, there was a significant group difference in the amount of gains observed the next day (i.e., motor sequence consolidation). Planned contrasts analyses also revealed that the Cond-NREM2 group exhibited greater gains in performance than the NoCond group (p = .006), and showed a trend toward a significant difference in gains compared to the Cond-REM group (p=.083). Again, as previously reported, performance of the Cond-REM and NoCond groups did not differ significantly (p = .27).

**Changes in sleep spindle characteristics bootstrap and multiple comparisons analyses**

Using this procedure for each of the spindle characteristics we found the following results: for spindle amplitude (NoCond>Cond-NREM2 in 6; Cond-NREM2>NoCond in 4994 cases out of 5000; see Figure S3), for spindle frequency (NoCond>Cond-NREM2 in 13; Cond-NREM2>NoCond in 4987 cases out of 5000; see Figure S4), for spindle duration (NoCond>Cond-NREM2 in 47; Cond-NREM2>NoCond in 4953 cases out of 5000), and for spindle density (NoCond>Cond-NREM2 in 3882; Cond-NREM2> NoCond in 1118 cases out of 5000). These results yielded the following p-values: 0.0012 (amplitude), 0.0026 (frequency), 0.0094 (duration) and 0.7764 (density). These findings are consistent with those reported in our manuscript. Moreover, they indicate that, using the described bootstrap procedure, there was a significant difference between the two groups (Cond-NREM2>NoCond) for amplitude and frequency, even after correcting for multiple comparisons (using Bonferroni correction for 12 comparisons: 4 characteristics*3 electrode locations – Pz, Cz, Fz; a reference p-value of 0.0041).

**Effect of categorizing sleep spindle frequencies at Pz**

The categorization algorithm used in this paper had the effect of reducing the overlap between frontal and parietal spindle frequencies. Further analyses were conducted to investigate the filtering effect on sleep spindle frequencies originating specifically at the Pz recording site. As expected, the median frequency at Pz in the *pre-matched* (without filtering = 13.428 Hz; with filtering = 13.428 Hz) and *during-stimulation* (without filtering = 13.428 Hz; with filtering = 13.428 Hz) sleep periods did not change whether filtering was applied or not. Yet the same categorization process did have the effect of slightly lowering the mean frequency in both *pre-matched* (without filtering = 13.3458 Hz; with filtering = 13.3307 Hz) and *during-stimulation* (without filtering = 13.3970 Hz; with filtering = 13.3823 Hz) sleep periods. Finally, the difference in frequency between the pre-matched and during-stimulation sleep periods were again similar whether filtering was applied or not (without filtering: 0.051 Hz; with filtering: 0.052 Hz). Hence, the categorization algorithm had no major impact on the frequency of Pz spindles during these sleep periods.

Analyses conducted on the filtered spindles dataset (and reported in the main article) were also ran on all spindles (before filtering). When we compared changes in spindle characteristics between the pre-matched and during-stimulation sleep periods, the one-way ANOVA comparing percent change (Δ%) revealed a significant difference in peak amplitude (F_1, 41_ = 4.950, p = .03) and a trend toward significance in peak frequency (F_1, 41_ = 3.043, p = .09) between the Cond-NREM2 and NoCond groups. Similar to results with the filtered spindles dataset, however, one-sample t-tests revealed that only the Cond-NREM2 group had a significant increase in Δ% in peak frequency (Cond-NREM2: t_20_ = 2.786, p = .01; NoCond: t_21_ = 1.308, p = .21) and Δ% in duration (Cond-NREM2: t_20_ = 5.667, p < .001; NoCond: t_21_ = -1.167, p = .23). Results for Δ% peak amplitude did not reach significance in either groups (Cond-NREM2: t_20_ = 1.993, p = .06; NoCond: t_21_ = -1.167, p = .26). Overall the results from unfiltered spindles are thus similar in many ways to the filtered ones (i.e., results presented in the main manuscript), although some effects were weaker. However, the differences found between both set of results highlight even more the importance of applying a categorization algorithm as a preprocessing step prior to analyzing sleep spindles.

**3. Supporting Reference:**

1. Lunneborg CE. Random assignment of available cases: Bootstrap standard errors and confidence intervals. Psychol Methods. 2001;6(4):402–12.
